# Self-assembled antioxidant enzyme-mimicking hydrogel: Targeting oxidative stress and macrophage organization for improving degenerated intervertebral discs

**DOI:** 10.1016/j.mtbio.2025.101586

**Published:** 2025-02-19

**Authors:** Yudong Fu, Hua Sun, Yongchao Jin, Shaohui Cheng, Yanyi Wu, Chen Liu, Lei Fan, Juqun Xi, Shixin Li, Liang Zhang

**Affiliations:** a Institute of Translational Medicine, School of Medicine, Yangzhou University, Yangzhou, Jiangsu, 225001, PR China; b Department of Orthopedics, Northern Jiangsu People's Hospital Affiliated to Yangzhou University, Yangzhou, Jiangsu, 225001, PR China; c College of Bioscience and Biotechnology, Yangzhou University, Yangzhou, Jiangsu, 225009, PR China; d School of Chemistry and Chemical Engineering, Yangzhou University, Yangzhou, Jiangsu, 225002, PR China; e The Key Laboratory of the Jiangsu Higher Education Institutions for Integrated Traditional Chinese and Western Medicine in Senile Diseases Control (Yangzhou University), Yangzhou, Jiangsu, 225001, PR China

**Keywords:** Hydrogels, Enzyme-mimicking activities, Intervertebral disc degeneration, Inflammation, Macrophage polarization

## Abstract

Intervertebral disc degeneration (IVDD) is a major contributor to lower back pain. At present, antioxidant therapy is regarded as one of the most promising strategies for treating IVDD, due to the critical role of reactive oxygen species (ROS) in its pathogenesis. Herein, we presented a self-assembled hydrogel, termed MnGAHs, formed through the crossing of manganese ions (Mn^2+^) and glycyrrheic acid (GA), which possessed the activities of antioxidant enzymes, including catalase (CAT) and superoxide dismutase (SOD). The obtained MnGAHs effectively scavenge ROS, reducing oxidative stress levels and alleviating the senescence of nucleus pulposus-derived mesenchymal stem cells (NPMSC), thereby mitigating IVDD. Furthermore, MnGAHs also promoted macrophage polarization towards M2 phenotype, reducing the inflammatory response and thereby inhibiting the progression of IVDD. By combining theoretical calculations with analyses of public databases, we revealed that the ROS-p53-p21 axis played a crucial role in the function of MnGAHs to reverse IVDD, a finding further confirmed by Western blot analysis. As a result, the injection of MnGAHs into the intervertebral disc (IVD) significantly alleviated the degeneration process in a rat model of puncture-induced IVDD. Therefore, the as-prepared antioxidant enzyme-mimicking hydrogels provide a promising and effective approach for treating IVDD.

## Graphical abstract

A self-assembled hydrogel, formed by the coordination of manganese ions and glycyrrhetinic acid, mimics the enzymatic activities of catalase (CAT) and superoxide dismutase (SOD), effectively targeting oxidative stress and promoting intervertebral disc regeneration.

**Figure:**
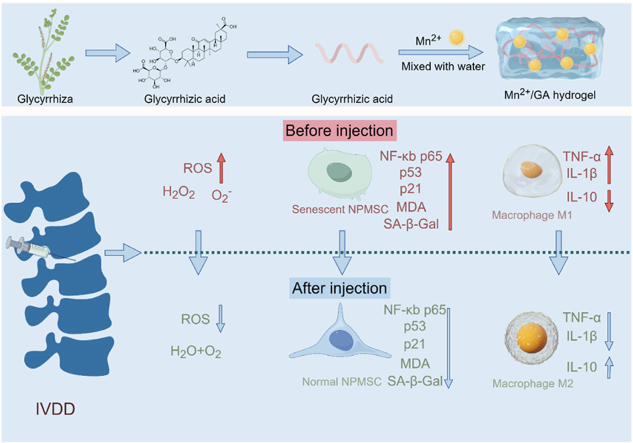

## 1 Introduction

Low back pain (LBP) significantly impacts patients’ quality of life, with approximately 80 % of individuals experiencing LBP at some point in their lives. As the aging population continues to grow, the incidence of LBP and its associated costs are rising rapidly [1,2]. The pathogenesis of LBP is complex, with spinal degenerative diseases resulting from intervertebral disc degeneration (IVDD), such as lumbar stenosis and spondylolisthesis, recognized as the primary causes of LBP [[3], [4], [5]]. The healthy intervertebral disc (IVD) is mainly composed of a surrounding annulus fibrosus (AF), a gelatinous nucleus pulposus (NP), and superior and inferior endplates (EPs) [[6], [7], [8], [9]]. In contrast, a degenerated IVD exhibits a significant decreased number and function in NP cells and abnormal breakdown of extracellular matrix (ECM) [[10], [11], [12]]. NP-derived mesenchymal stem cells (NPMSC) which was first discovered in human degenerative NP tissue in 2007 provides a new direction for the etiological study of IVDD [13]. It has been found that NPMSC are the main source of endogenous NP cells in NP, which promote the repair and regeneration of degenerative IVDD by differentiating into NP cell and/or inhibiting the apoptosis of NP cell [[14], [15], [16]]. Previous studies have confirmed that endogenous NPMSC are present in both normal and degenerated IVD and possess multi-directional differentiation potential [17,18]. However, an unfavorable microenvironment leads to aging and apoptosis of NPMSC, which is believed as a major feature of IVDD. Due to the complex molecular mechanisms underlying IVDD, current clinical treatment options, including analgesics, anti-inflammatory medications, and physical therapy, as well as surgical interventions considered as a last resort, are limited. These approaches can only partially alleviate clinical symptoms and do not address the underlying physiological and pathological processes of IVDD [19,20]. Thus, this situation highlights the importance of finding an effective treatment strategy for managing IVDD.

Previous research has shown a strong correlation between IVDD and the excessive production of reactive oxygen species (ROS) within the microenvironment of the IVD [21]. Elevated levels of ROS, such as hydroxyl radical (•OH), hydrogen peroxide (H_2_O_2_), superoxide anion (O_2_^•−^), and peroxynitrite (ONOO^−^), can induce oxidative stress. Importantly, the oxidative stress triggered by over-produced ROS not only promotes the lipid peroxidation, protein oxidation, and DNA damage but also intensifies inflammation, causing the death of functioning cells during IVDD [22]. Oxidative stress can also lead to the decline of proliferation, migration and differentiation of NPMSC [18,23]. This condition may lead to the failure of the endogenous repair process. Therefore, it is particularly important to maintain the number of viable and functional NPMSC during regeneration of IVDD. Furthermore, macrophages are the only inflammatory cells able to infiltrate the closed NP tissue. The quantity of macrophages is positively correlated with the severity of IVDD, particularly M1 macrophages—commonly known as “classical” macrophages—which play a crucial role in the inflammatory response and the secretion of pro-inflammatory cytokines, thereby further worsening IVDD degradation [[24], [25], [26], [27], [28], [29]]. Importantly, ROS as an important mediator can regulate the response of macrophages through both direct and indirect mechanisms [[30], [31], [32], [33]]. Therefore, we hypothesize that eliminating excess ROS and simultaneously maintaining the balance of M1-M2 macrophage polarization in IVD environment may represent a promising strategy for effectively treating IVDD.

Nanozymes are a new class of nanocatalysts that exhibit catalytic activity and enzymatic reaction kinetics akin to those of natural enzymes [[34], [35], [36]]. The catalytic functions of nanozymes are crucial for various therapeutic applications. For instance, nanozymes exhibiting superoxide dismutase (SOD) and catalase (CAT) activity can effectively scavenge ROS to protect cells [37]. In contrast, those with peroxidase (POD) and oxidase (OXD) activity can generate ROS to eliminate harmful cells, such as tumor cells and bacteria [38,39]. Considering the relationship between ROS and IVDD, the use of antioxidant nanozymes may represent a promising strategy for intervening in IVDD. However, achieving targeted delivery of nanozymes to specific sites within the IVD poses significant challenges. Factors such as particle size, surface charge, and degradation rates can greatly influence the distribution and retention of nanozymes in the body [40]. Consequently, identifying an appropriate vehicle for nanozyme delivery is essential. Hydrogels, as widely utilized biomaterials, have shown great promise for treating IVDD due to their ability to mimic the mechanical properties of mammalian tissues, provide mechanical support to the 10.13039/100006209NP, and enable minimally invasive delivery of encapsulated drugs or genes for targeted treatment of IVDD. Therefore, we aimed to develop a hydrogel with antioxidant enzyme-mimicking activities to improve IVDD treatment outcomes.

Glycyrrhizic acid (GA), the main active component of licorice root, has been recognized in traditional Chinese medicine for centuries due to its wide range of bioactivities, including anti-tumor, anti-inflammatory, anti-ulcer effects [41,42]. Because of its amphiphilic nature, GA can form self-assemblies in both aqueous and non-aqueous environments [43]. Specifically, polyvalent metal ions such as Ca^2+^, Cu^2+^, and Zn^2+^ can enhance the self-assembly of glycyrrhizic acid (GA) into structures like micelles, vesicles, and hydrogels. Thus, incorporating antioxidant enzyme activity into the GA-based hydrogel forms the foundation of our work here. Considering the structure of SOD, Mn-SOD, the third isoform, consists of a homotetramer, with each of the four subunits containing an active site that includes manganese as a cofactor [44]. Therefore, we propose that the structure resulting from the interaction between Mn ions and GA molecules may simulate the antioxidant activity of Mn-SOD. In this model, Mn ions mimic the catalytic cofactor of Mn-SOD, while the amphiphilic structures of GA replicate the microenvironment surrounding the active center.

**Scheme 1:**
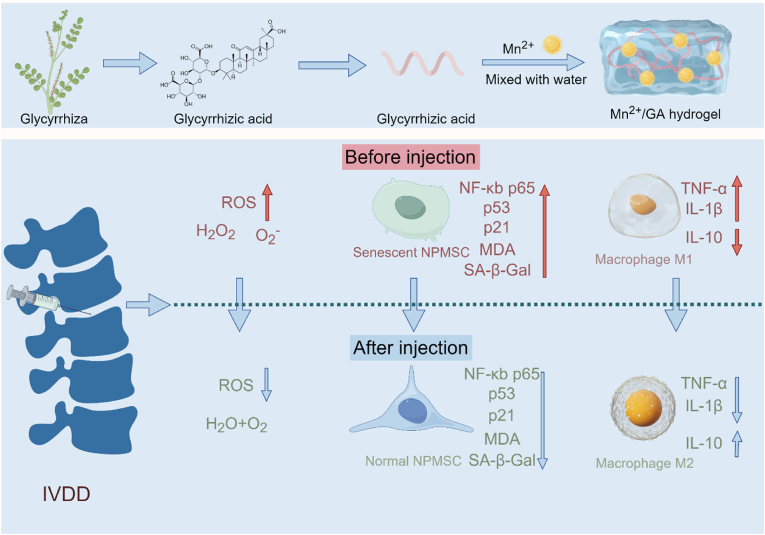
Schematic diagram illustrating the preparation of Mn^2+^/GA hydrogel and its mechanism for ameliorating IVDD (drawn by Figdraw).

In this work, we developed a novel injectable multipurpose hydrogel, formed by crosslinking Mn ions with GA molecules, which we refer to as Mn^2+^/GA hydrogels (MnGAHs). The gelation process and underlying driving forces were validated through a series of methods, including molecular docking, isothermal titration microcalorimetry (ITC), rheological and spectral analysis. This as-prepared MnGAHs exhibited antioxidant properties by co-mimicking the cofactor and microenvironment of natural enzymes. At the cellular level, MnGAHs can reduce oxidative stress and alleviate the senescence of NPMSC. Additionally, MnGAHs promote macrophage polarization toward the M2 phenotype. By combining theoretical calculations with analyses of public databases, we revealed that the ROS-p53-p21 axis played a crucial role in the function of MnGAHs to reverse IVDD, a finding further confirmed by Western blot (WB) result (Scheme 1). Furthermore, we validated the therapeutic efficacy of MnGAHs using a rat model of puncture-induced IVDD, confirmed through Magnetic Resonance Imaging (MRI), as well as immunofluorescence and histochemical assessments. Consequently, our work presents a novel therapeutic candidate based on self-assembled antioxidant enzyme-mimicking hydrogels for IVDD therapy.

## 2 Results and discussion

### 2.1 Design and characterization of MnGAHs

**Fig. 1:**
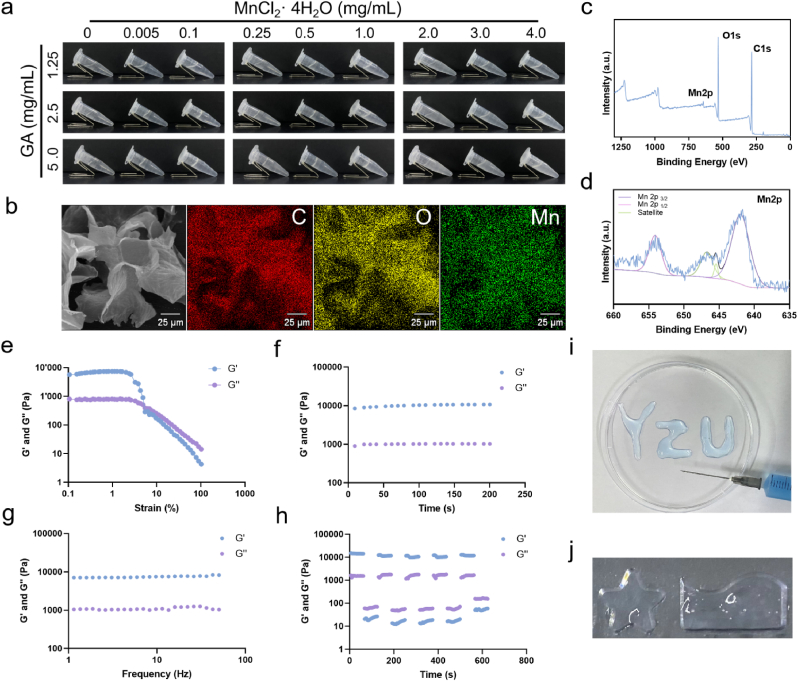
Design and characterization of MnGAHs. **(a)** Representative optical images of MnGAHs prepared under different concentrations of MnCl_2_ and GA. **(b)** SEM image and corresponding elemental mapping of MnGAHs. **(c, d)** XPS patterns of MnGAHs, including the deconvolution of Mn2p. **(e**–**g)** Rheological analysis of MnGAHs as a function of shear strain (e), time (f), and frequency (g). **(h)** Damage-healing properties of MnGAHs presented through continuous step strain measurements (0.5 % strain → 40 % strain → 0.5 % strain) at a fixed time interval of 120 s. **(i, j)** Images illustrating the injectability and moldability of MnGAHs.

In this study, we developed a multifunctional hydrogel to ameliorate IVDD, which was fabricated through the interactions between metal ions and herbal molecules. Fig. 1a illustrated the Eppendorf tubes containing GA solutions with and without MnCl_2_. The tubes were placed vertically on their caps for 10 min (steady-state) to demonstrate that gelation occurred at specific concentrations of GA and MnCl_2_. However, the control sample containing only GA remained a low-viscosity liquid without gelation. According to Fig. 1a and Table S1, the gelation occurred within a range of MnCl_2_ concentrations between 0.1 and 1.0 mg/mL relative to GA at 5 mg/mL, underscoring the critical importance of MnCl_2_ concentration for gelation. Importantly, the hydrogels containing 5 mg/ml GA and 1 mg/mL Mn^2+^ could be rapidly formed within 1 min (Fig. S1). Scanning electron microscopy (SEM) was then used for observing the microstructural characteristics of the crosslinked hydrogels (Fig. 1b), revealing a porous structure in lyophilized MnGAHs synthesized at 5 mg/mL GA and 1 mg/mL Mn^2+^. Additionally, elemental mapping of the MnGAHs in Fig. 1b confirmed the presence of carbon (C), oxygen (O), and manganese (Mn), demonstrating the successful introduction of Mn ions into the hydrogels. Furthermore, the result X-ray Photoelectron Spectroscopy (XPS) clearly showed the coexistence of Mn2p (640.0 eV), C1s (283.1 eV), and O1s (532.0 eV) peaks in MnGAHs (Fig. 1c), further verifying the assembly of GA with Mn ions [45]. As expected, the high-resolution XPS spectra of Mn2p (Fig. 1d) displayed two peaks at 652.6 and 642.1 eV, corresponding to Mn (2p1/2) and Mn (2p3/2), respectively, with a Mn atom content of 7.61 % in the hydrogels [34]. These results indicated that Mn^2+^ can stimulated the GA solution to occur gelatin, yielding of a composite hydrogel.

Next, the theological test was performed on MnGAHs to evaluate its self-healing properties. First, the response behaviors of MnGAHs to external strains were investigated using rheological strain sweep measurements. As the applied strain rose from 0.1 % to 1 %, the storage modulus (G′) and loss modulus (G'') of MnGAHs remained constant (Fig. 1e). When the strain was larger than 1 %, a drop of G′ value was found, showing a crossover point with G” at 5 % strain. This phenomenon suggested that the hydrogel network was ruptured at this strain, resulting in a transition from the gel state to the sol state. Furthermore, the time sweep measurements in Fig. 1f revealed that the values of G′ consistently exceeded those of G″ over the 200 s testing period, indicating the stability of MnGAHs [46]. The frequency-dependent time rheological behavior with the range of 1–50 Hz was evaluated through oscillating frequency measurements. As shown in Fig. 1g, the MnGAHs consistently maintained its colloidal morphology throughout the frequency range. Next, a continuous step change in oscillatory strain between 0.5 % and 40 % at a frequency of 10 Hz was employed to evaluate the strain-induced damage and healing of the MnGAHs. As shown in Fig. 1h, a large dynamic strain of 40 % reduced G′ to approximately 20 Pa, indicating structural damage to the hydrogel. However, the G′ value quickly returned to its initial level after a holding period at a lower strain of 0.5 %, demonstrating the successful recovery of the hydrogel's structure. Importantly, MnGAHs exhibited robust and repeatable restoration of their rheological properties following the continuous step strain, indicating their ability to spontaneously recover their structure after damage. Next, an extrusion test was conducted (Fig. 1i), in which the letters “YZU” (representing “Yangzhou University”) were injected using a syringe with a 0.5 mm needle, demonstrating that MnGAHs have good injectability. The shape of the hydrogel can be controlled by injecting it into different molds, indicating that MnGAHs possess excellent moldability (Fig. 1j). Furthermore, MnGAHs have low swelling and degradation rates (Fig. S2).

### 2.2 Driving forces in gelation of MnGAHs

**Fig. 2:**
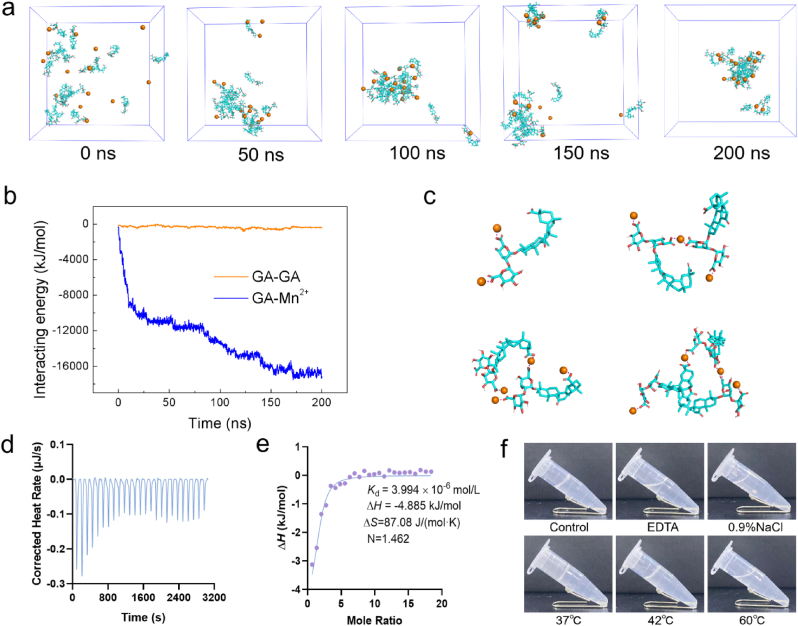
Analysis of dominant interactions in the assembly of MnGAHs. **(a)** Time evolution of representative snapshots from the molecular dynamics simulation. **(b)** Time evolution of the interaction energy for GA-GA and GA-Mn^2+^ interactions. **(c)** Molecular details of the assembled structure. **(d)** ITC measurement and **(e)** binding isotherm for the titration of MnCl_2_ into the GA solution. **(f)** Optical images of MnGAHs under various conditions.

To better illuminate the driving forces during the assembly process, molecular dynamics simulations were performed to investigate the assembly structures of GA molecules and Mn^2+^ at the molecular level. As shown in Fig. 2a, GA molecules and Mn^2+^ could spontaneously assemble into an aggregated state, facilitated by the increased interaction between GA molecules and Mn^2+^ (Fig. 2b). Notably, the interaction energy between GA molecules was close to zero, highlighting the important role of Mn^2+^ in the assembly process. Typical molecular details of assembly structure were depicted in Fig. 2c, where Mn^2+^ formed salt bridges with carboxyl groups of GA molecules, serving as the primary connection between them. Then, Fourier transform infrared (FTIR) spectroscopy was further utilized to perform a structural investigation. As shown in Fig. S3, GA exhibited a peak at 3426 cm^−1^, corresponding to the stretching vibration of –OH groups, a peak at 1733 cm^−1^ for the stretching vibration of C=O groups, and a peak at 1648 cm^−1^ for the aromatic skeleton vibration of C=C bonds. Furthermore, the peak at 1459 cm^−1^ was attributed to the vibration of aromatic C–C stretching, while the peak at 1045 cm^−1^ was assigned to the vibration of C–O. In the FTIR spectrum of MnGAHs, the notable decrease in the intensity of the C=O peak suggested that the interactions between Mn^2+^ and the carboxyl groups of GA were the primary driving force behind the formation of MnGAHs. We then employed isothermal titration calorimetry (ITC) to detect the heat associated with the binding reaction between Mn^2+^ and GA. From these experiments, we can directly determine the enthalpy, equilibrium constant, and reaction stoichiometry for the binding reaction. This data allows us to calculate the Gibbs free energy and the change in entropy using the following equations: Δ*G*° = −*RT*ln*K* and Δ*G*° = Δ*H*−*T*Δ*S*. As shown in Fig. 2d and e, the entropy change for Mn^2+^ binding with GA was positive (87.08 J/(mol⋅K)), and the negative Gibbs free energy (−30.84 kJ/mol) showed that the adsorption of Mn^2+^ onto GA molecules was spontaneous. Additionally, the dissociation constant (*K*_d_) and reaction stoichiometry for Mn^2+^ binding to GA were found to be 3.994 × 10^−6^ mol/L and 1.462, respectively, confirming the binding interaction between Mn^2+^ and GA. Next, we treated the MnGAHs with NaCl and ethylenediaminetetraacetic acid (EDTA). EDTA chelates Mn^2+^, disrupting the salt bridges formed with the carboxyl groups of GA molecules, while NaCl interferes with the ionic interactions. As shown in Fig. 2f, NaCl and EDTA disrupted the dominant stabilizing forces, resulting in increased fluidity of the system. Meanwhile, an increase in temperature also weakened the interaction between Mn^2+^ and GA. Together, the theoretical calculations, ITC, and FTR experiments all confirmed that the dominant interactions in the assembly of MnGAHs arise from the formation of salt bridges.

### 2.3 Mimicking antioxidant enzymes to alleviate cellular oxidative stress

**Fig. 3:**
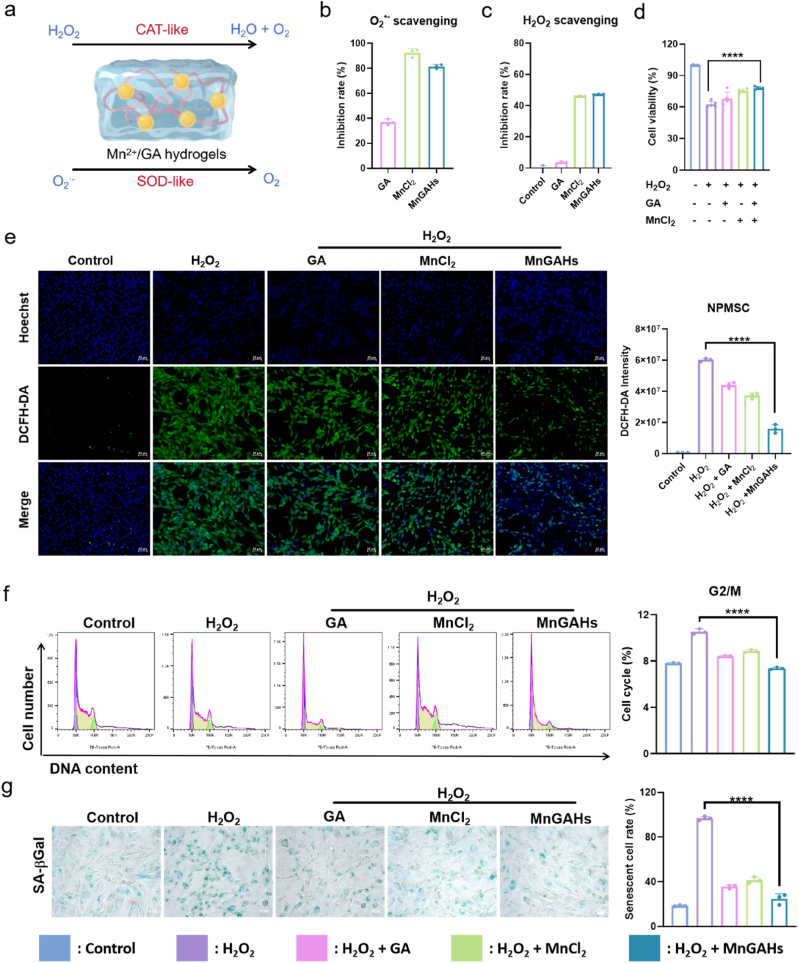
Behavior of NPMSC cultured with MnGAHs. **(a)** Schematic representation of MnGAHs exhibiting SOD- and CAT-like activities (drawn by Figdraw). **(b)** SOD-like activity of MnGAHs (n = 3). **(c)** CAT-like activity of MnGAHs (n = 3). **(d)** Cell viability of NPMSC following various treatments (n = 5). **(e)** ROS levels (measured by the DCFH-DA assay) and corresponding statistical results in NPMSC after incubation with H_2_O_2_, followed by treatments with GA, MnCl_2_, and MnGAHs, respectively. The nucleus was stained with Hoechst (Scale bar = 50 μm, n = 3). **(f)** Effect of MnGAHs on cell cycle progression and corresponding statistical results in NPMSC, as assessed by flow cytometry (n = 3). **(g)** Representative images of β-galactosidase staining and corresponding statistical results of positive cells (Scale bar = 200 μm, n = 3). ∗∗∗∗P < 0.0001.

Next, the ROS scavenging activities of MnGAHs were evaluated. The representative ROS chosen to assess the antioxidant capacity of MnGAHs included O_2_^⋅-^ and H_2_O_2_ (Fig. 3a). As shown in Fig. 3b, compared to GA alone, MnCl_2_ exhibited a significant ability to scavenge O_2_^⋅-^. MnGAHs retained the capacity of Mn^2+^, demonstrating SOD activity. Additionally, MnGAHs exhibited CAT activity, decomposing H_2_O_2_ into O_2_. To better illuminate the primary source of the enzyme-like activity of MnGAHs, we tested the ROS scavenging activities of MnCl_2_. As demonstrated in Fig. S4, MnCl_2_ exhibited a dose-dependent manner in the clearance of O_2_^⋅-^, with 90 % of O_2_^⋅-^ being scavenged by 21 μg/mL MnCl_2_. MnCl_2_ can simultaneously generated O_2_ from H_2_O_2_, demonstrating its capability as a CAT enzyme (Fig. 3c). These results indicated that the Mn^2+^ in MnGAHs served as the catalytic center, endowing the hydrogels with enzyme-mimicking activities like those of natural enzymes [47]. Collectively, MnGAHs exhibited SOD and CAT activities, allowing them to effectively scavenge ROS.

To evaluate the therapeutic effects of MnGAHs on IVDD form a cellular perspective, H_2_O_2_ was used to induce NPMSC oxidative stress. The cell viability assay (Fig. 3d) suggested a decreased survival rate following H_2_O_2_ treatment compared to the control group. Treatment with the MnCl_2_ or GA alone in certain concentration range improved the cell survival rate owing to its excellent antioxidant property (Fig. S5). Importantly, the hydrogels composed of MnCl_2_ and GA demonstrated a greater cellular protective effect compared to the individual components (Fig. 3d). This result was also shown by the Calcein AM (live)/PI (dead) double staining results. The results in Fig. S6 showed that the percentage of PI-positive NPMSC significantly increased after H_2_O_2_ treatment compared to the control group. Flow cytometry results in Fig. S7 also showed that the apoptosis rate of NPMSC treated with MnGAHs was significantly lower than that of H_2_O_2_-induced group. However, H_2_O_2_-induced cell oxidative stress was attenuated after preincubated with MnGAHs. Next, the protective effect of MnGAHs against oxidative stress was further investigated *in vitro* using the fluorescence probe 2′,7′-dichlorofluorescin diacetate (DCFH-DA). This probe can be oxidized by intracellular ROS to produce 2′,7′-dichlorofluorescein (DCF), which emits green fluorescence. NPMSC treated with H_2_O_2_ were used as a positive control to elevate intracellular ROS levels. As illustrated in Fig. 3e, significant green fluorescence was detected in cells treated with H_2_O_2_, which was alleviated by MnGAHs. The oxidative index product, malondialdehyde (MDA), showed a similar trend, with the lowest levels observed after MnGAHs treatment in H_2_O_2_-stimulated NPMSC (Fig. S8). All these results confirmed the exceptional intracellular ROS scavenging activity of MnGAHs. We then investigated whether MnGAHs could rescue G2/M cell cycle arrest *in vivo*. NPMSC from different groups were collected and analyzed for cell cycle distribution using flow cytometry (Fig. 3f). The proportion of cells arrested in the G2/M phase was less than 8 % in the control group. In the H_2_O_2_-treated group, this proportion increased to approximately 11 % but decreased back to about 8 % after MnGAHs treatment. To further explore the protective effect of MnGAHs on NPMSC, we used H_2_O_2_ to induce cellular senescence. As shown in Fig. 3g, H_2_O_2_ treatment resulted in increased expression of senescence-associated β-galactosidase (SA-β-gal), which is a notable sign of senescence. However, treatments with GA and MnCl_2_ reduced SA-β-gal expression, with MnGAHs further enhancing this effect. Collectively, these results suggest that the self-assembled MnGAHs, which possess SOD- and CAT-like activities, significantly protect NPMSC from H_2_O_2_-induced oxidative stress.

### 2.4 Regulating macrophage polarization to mitigate cellular inflammation

**Fig. 4:**
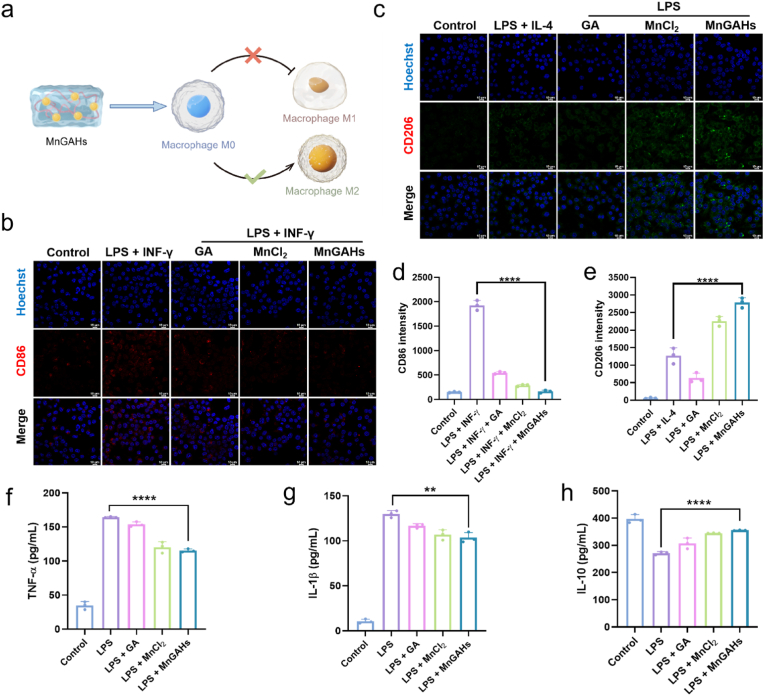
Immunomodulatory activity of MnGAHs. **(a)** Schematic diagram of the experiment to study the polarizing effect of MnGAHs on M0 macrophages (drawn by Figdraw). **(b, c)** Representative immunofluorescence images, and **(d, e)** their corresponding quantification analysis of macrophage phenotypes in different groups (Scale bar = 10 mm, n = 3). Macrophages were stained with CD86 **(b)** in red or CD206 **(c)** in green, while nuclei were stained with Hoechst in blue. **(f**–**h)** Levels of cytokines including **(f)** TNF-α, **(g)** IL-1β, and **(h)** IL-10, in the RAW264.7 cells were measured by ELISA (n = 3). ∗∗P < 0.01; ∗∗∗∗P < 0.0001.

Inflammation modulation is a crucial aspect of IVDD. Regulating macrophage polarization is a common strategy to inhibit the inflammatory response. Notably, macrophages are the only inflammatory cells capable of infiltrating the closed NP tissue. M1 macrophages are associated with inflammation, while M2 macrophages play a role in tissue healing. Therefore, we next investigated the effect of MnGAHs on macrophage polarization (Fig. 4a). In Brief, we treated RAW264.7 cells (mouse mononuclear macrophage leukaemia cells) with MnGAHs, as well as MnCl_2_ and GA for comparison. First, consistent with the results observed in NPMSC, MnGAHs also decreased ROS levels in H_2_O_2_-treated RAW264.7 cells (Fig. S9). Then, Fig. 4b and c illustrated macrophage infiltration, evaluated through immunofluorescence staining. M1 and M2 macrophages were identified by the markers CD86 and CD206, respectively [48]. Compared to the untreated group and those treated with MnCl_2_ or GA, MnGAHs increased the proportion of CD206-positive macrophages and significantly decreased the proportion of CD86-positive macrophages, indicating a shift toward a less inflammatory state. The corresponding statistical data for fluorescence intensities were shown in Fig. 4d and e. Thus, MnGAHs effectively promoted macrophage polarization toward the anti-inflammatory M2 phenotype, which would lead to increased levels of anti-inflammatory cytokines. As expected, Fig. 4f–h demonstrated that lipopolysaccharide (LPS) treatment significantly increased the levels of M1-associated cytokines (TNF-α, IL-1β) while reducing the levels of M2-associated cytokines (IL-10). Importantly, TNF-α levels in LPS-treated group rose by approximately 4-fold relative to the control group, suggesting excessive inflammation in RAW264.7 cells, which was significantly mitigated by MnGAHs treatment. Additionally, the expression of the anti-inflammatory cytokine IL-10 was notably higher in the MnGAHs group compared to the other groups. Therefore, MnGAHs can promote M1-to-M2 polarization in a cellular inflammation model.

### 2.5 Revealing the mechanism of MnGAHs

**Fig. 5:**
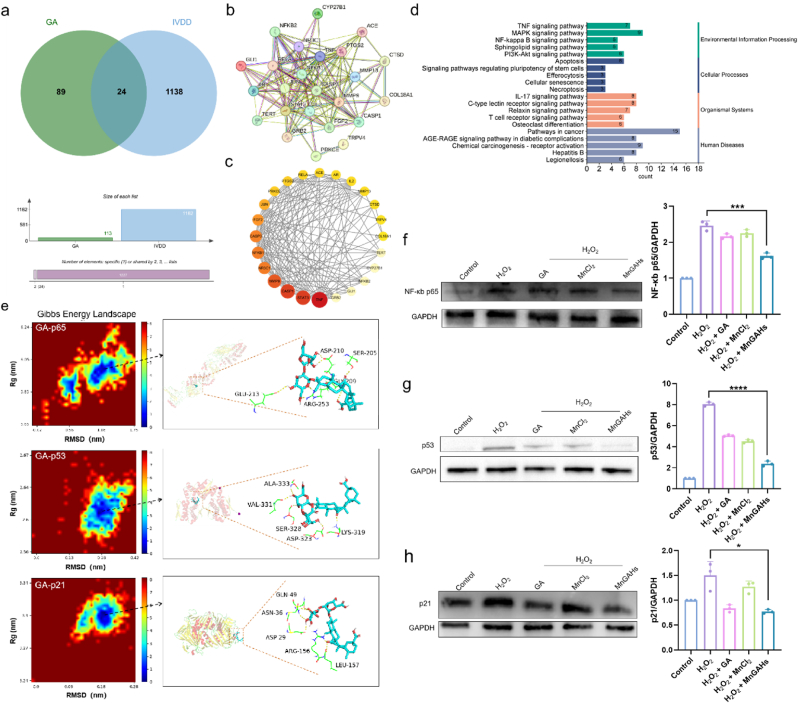
Mechanism by which MnGAHs ameliorated IVDD. **(a)** Venn diagram illustrating the potential targets of GA against IVDD. **(b)** Protein-protein interaction network depicting 24 co-targets of GA for IVDD therapy. **(c)** Network of target genes generated using Cytoscape, where nodes represented target genes, with darker colors indicating greater importance. **(d)** Results of KEGG enrichment analysis. **(e)** Binding modes of GA with p65, p53, and p21. Left: distributions of Gibbs free energy landscapes based on simulation trajectories. Right: visualization of the binding conformations, with dashed lines representing hydrogen bonds. **(f**–**h)** Western blot analysis of p65, p53, and p21, along with corresponding statistical results (n = 3). ∗P < 0.05; ∗∗∗P < 0.001; ∗∗∗∗P < 0.0001.

To further explore the mechanism by which MnGAHs alleviate IVDD, we employed molecular docking and network pharmacology to predict potential targets and pathways of GA in IVDD treatment. Initially, we retrieved 1162 targets associated with IVDD and 113 targets related to GA from various databases. Cross-analysis identified a total of 24 potential GA targets for IVDD (Fig. 5a). To investigate protein-protein interactions, the data for these 24 targets was subsequently submitted to the Search Tool for the Retrieval of Interacting Genes/Proteins (STRING) database (Fig. 5b). We established the confidence level at 0.4 and created a protein-protein interaction (PPI) network comprising 24 nodes and 140 edges. Then, we imported it into Cytoscape 3.10.0 and gained the network where the nodes represented target genes, and the size and color of each node represented its level of betweenness. The central properties of the nodes were estimated by topological analysis with CytoNCA (Fig. 5c). Notably, TNF, STAT3, CASP1, MMP9, NR3C1, NFKB1, and MMP13 were the potential core targets for GA treatment of IVDD in the PPI network. Subsequently, Gene Ontology (GO) enrichment analysis showed that these targets were involved in biological processes, including the positive regulation of transcription by RNA polymerase II, negative regulation of transcription by RNA polymerase II, positive regulation of miRNA transcription, positive regulation of DNA-templated transcription, and apoptotic processes, among others (Fig. S10a). Regarding cellular components, the targets were predominantly enriched in the cytoplasm, nucleus, cytosol, nucleoplasm, and the NF-κB p50/p65 complex, among others (Fig. S10b). In terms of molecular functions, these targets are mainly linked to identical protein binding, sequence-specific DNA binding to RNA polymerase II cis-regulatory regions, transcription cis-regulatory region binding, DNA-binding transcription factor activity, and RNA polymerase II-specific DNA-binding transcription activator activity, among others (Fig. S10c). The Kyoto Encyclopedia of Genes and Genomes (KEGG) enrichment analysis demonstrated that these targets were strongly associated with apoptosis, cellular senescence, the TNF signaling pathway, the NF-κB signaling pathway, the T cell receptor signaling pathway, and various cancer-related pathways, among others (Fig. 5d). As demonstrated by the results of GO and KEGG enrichment analysis, we chose three key targets: p65, p53 and p21. The p65 is a key target in the NF-κB pathway and p53 plays an important role in apoptosis, cell senescence and the NF-κB pathway. Meanwhile, p21 is involved in both cellular senescence and DNA-templated transcription.

Based on the results of network pharmacology, we utilized simulations of molecular dynamics and molecular docking to explore the binding modes of GA with target protein, including p65, p53, and p21. During 50 ns molecular dynamics (MD) simulations, the root means square displacement (RMSD) for GA and these proteins remained small (Figs. S11a and S11c), indicating that the binding modes of docking complex are stable. This stability was further confirmed by the consistent radius of gyration (*R*g) observed throughout the 50 ns simulations (Fig. S11d-S11f). Based on the simulation trajectories, we constructed the Gibbs Energy Landscape and visualized the binding modes corresponding to the lowest Gibbs Energy states (Fig. 5e). For the p65 protein, stable hydrogen bonds with GA were observed with Glu213, Asp210, Ser205, Gly209, and Arg253. In the case of the p53 protein, stable hydrogen bonds were found with Ala333, Val331, Ser328, Asp323, and Lys319. Additionally, GA formed stable hydrogen bonds with Gln49, Asn36, Asp29, Arg156, and Leu157 in the p21 protein. We then investigated key signaling pathways, including the p65, p53, and p21 pathways. Western blot analysis revealed that the p65 signaling pathway was activated following H_2_O_2_ treatment. However, co-treatment with MnGAHs inhibited this pathway (Fig. 5f). We therefore hypothesized that MnGAHs exert their effects by modulating the NF-κB pathway. To further explore the role of MnGAHs, we also examined the p53 and p21 signaling pathways, obtaining similar results (Fig. 5g and h). Based on our theoretical calculations and experimental findings, we conclude that the GA-based hydrogels can modulate the inflammatory response by inhibiting the p65, p53, and p21 signaling pathways.

### 2.6 Therapeutic efficacy of MnGAHs in a rat model of puncture-induced IVDD

Before assessing the therapeutic effects of MnGAHs in alleviating IVDD, we first evaluated their biosafety. The cell viability of NPMSC was quantitatively measured using the thiazolyl blue tetrazolium bromide (MTT) assay, demonstrating that MnGAHs exhibited comparable cell viability to the control group (Fig. S12), with cell viability levels exceeding 80 %. Additionally, the effect of MnGAHs on NPMSC migration was observed through scratch experiments. The migration rates of NPMSC treated with MnGAHs differed from the control groups, with the highest migration rate recorded in the MnGAH-treated group. Quantitative analysis of the scratch area showed that the 24-h scratch healing ratio in the MnGAH-treated group was approximately 250 %, higher than that in the MnCl_2_-treated group (200 %) and GA-treated group (137 %) (Fig. S13), indicating that MnGAHs significantly promoted cell migration. The hemolysis ratio of MnGAHs was determined to be 4.3 % (Fig. S14). Biosafety evaluations were also conducted in healthy rats administered MnGAHs. Standard hematological tests were conducted, as shown in Fig. S15, and revealed no significant changes in blood cell counts or biochemistry following 7 days of MnGAHs administration. Serum levels of alanine transaminase (ALT), alkaline phosphatase (ALP), aspartate transaminase (AST), blood urea nitrogen (BUN), and creatinine (CREA) measured 7 days post-injection showed no noticeable differences among all groups, indicating that MnGAHs posed no apparent toxicity to the liver or kidneys. Furthermore, there were no significant differences in blood parameters, including red blood cells (RBC), hematocrit (HCT), hemoglobin (HGB), mean corpuscular volume (MCV), and mean corpuscular hemoglobin (MCH) throughout the study period (Fig. S16), suggesting a normal biochemical status. Additionally, Hematoxylin and Eosin (H&E)-stained sections of the heart, liver, spleen, lung, and kidney from rats treated with MnGAHs showed no signs of tissue or cellular damage (Fig. S17). These findings indicate that MnGAHs are biocompatible and well-tolerated in healthy rats.

**Fig. 6:**
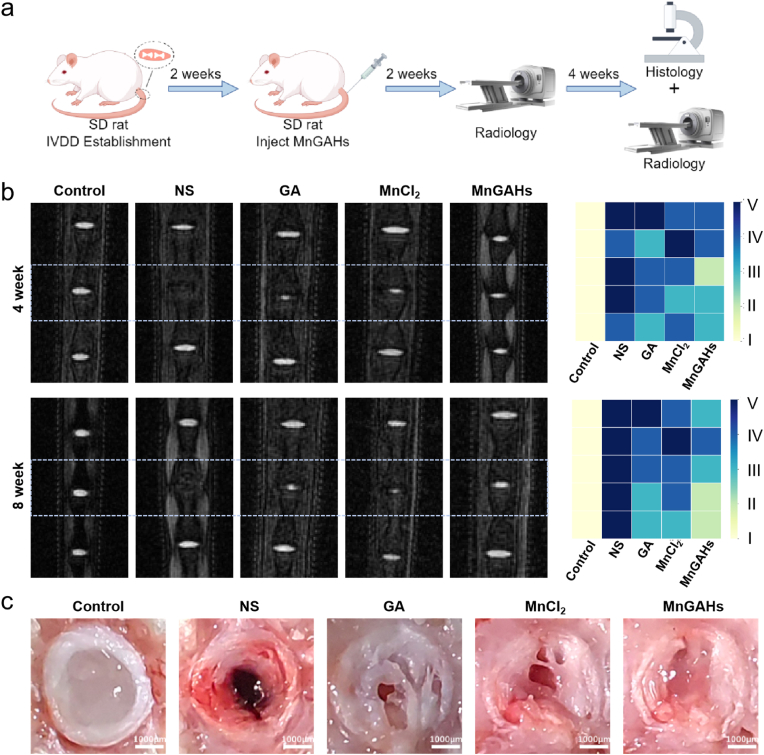
Effect of MnGAHs in the rat IVDD model. **(a)** Schematic representation of the experiment designed to investigate the effects of MnGAHs in the IVDD rat model (drawn by Figdraw). **(b)** MRI images (T2-weighted imaging, T2WI) of the rat caudal vertebrae taken after 4 and 8 weeks of modeling. The degenerative grades of IVDs were evaluated using the Pfirrmann grading system (n = 5). **(c)** Representative intervertebral disc photos from different groups. (Scale bar = 1000 μm).

**Fig. 7:**
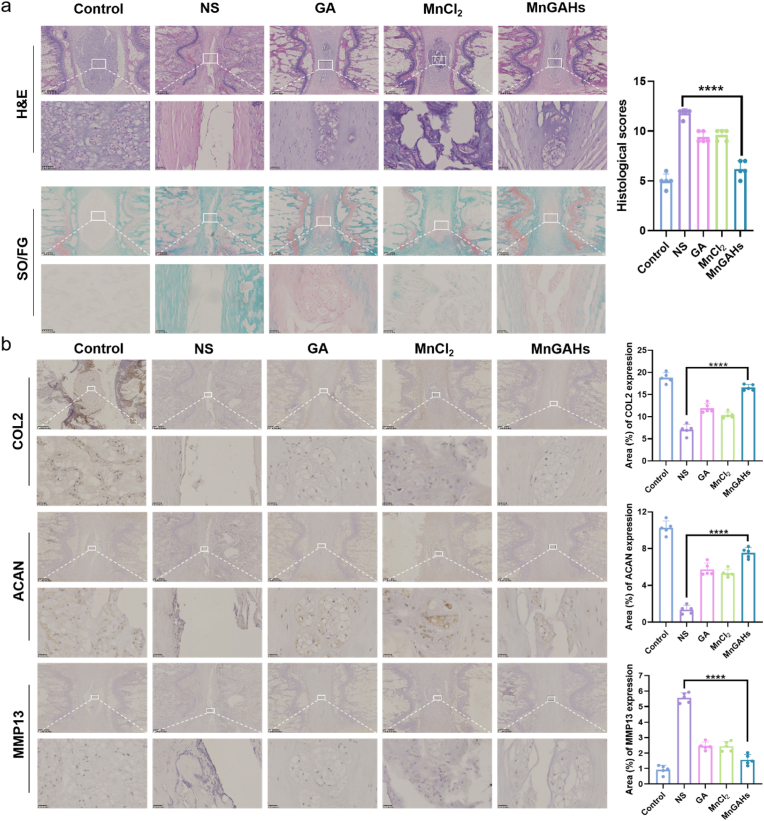
Histological and immunohistochemistry analysis. **(a)** H&E and SO/FG staining of the operated segments (Scale bar = 500 μm, and 50 μm for enlarged images, n = 5). **(b)** Immunohistochemistry images in each group of rat IVD tissues, including COL2, ACAN and MMP13, along with corresponding statistical results. (Scale bar = 500 μm, and 25 μm for enlarged figures). ∗∗∗∗P < 0.0001.

Subsequently, we conducted animal experiments (Fig. 6a). All procedures were approved by the Laboratory Animal Ethics Committee of Yangzhou University. Forty male Sprague-Dawley rats (weighing 200–220 g and aged 2–3 months) were obtained from the Experimental Animal Center of Yangzhou University and randomly assigned into 5 groups: (I) Control group (non-puncture), (II) NS group (puncture + normal saline (NS) injection), (III) GA group (puncture + GA injection), (IV) MnCl_2_ group (puncture + MnCl_2_ injection), and (V) MnGAHs group (puncture + MnGAHs injection). As depicted in Fig. 6b, the T2-weighted signal intensity in the NS group decreased significantly at the 4th week post-surgery and completely disappeared by the 8th week. In contrast, the T2-weighted signal intensity in the GA, MnCl_2_, and MnGAHs groups was markedly higher than that in the NS group at both 4 and 8 weeks after treatment, with the MnGAHs group showing the most significant improvement. Pfirrmann grading analysis of each group indicated that MnGAHs treatment effectively prevented IVD degradation. At the same time, the results were also reflected in the representative intervertebral disc photos from different groups of SD rats (Fig. 6c). Additionally, H&E staining and Safranin O/Fast Green (SO/FG) staining (Fig. 7a) revealed significant disruption of the boundary between NP and AF in the IVD of the NS group, with nearly complete loss of NP organization and replacement of red-stained proteoglycans by green collagenous structures over time. By 8 weeks, the IVD structures in the GA and MnCl_2_ groups exhibited varying degrees of damage but were still better preserved compared to the NS group. In contrast, MnGAHs treatment substantially ameliorated NP and AF degradation and morphological changes, showing abundant red proteoglycan staining in NP tissues and relatively organized AF structures. Histological scoring at week 8 showed the highest grade in the NS group, indicating severe IVDD, whereas the MnGAHs group demonstrated a significant reduction in histological grade, underscoring the efficacy of MnGAHs treatment in improving IVDD (Table S3).

**Fig. 8:**
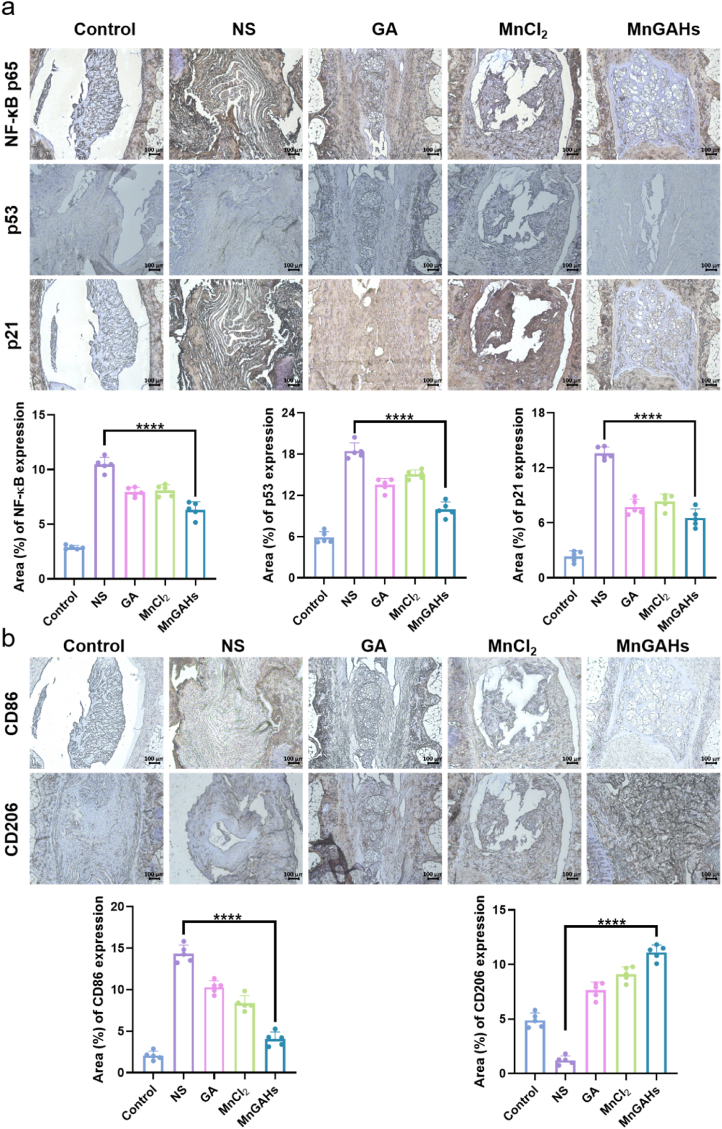
Immunohistochemistry analysis. Immunohistochemistry images in each group of rat IVD tissues and corresponding statistical results, including NF-κB p65, p53, p21 **(a)**, CD86 and CD206 **(b)**. (Scale bar = 100 μm, n = 5) ∗∗∗∗P < 0.0001.

The 8-week immunohistochemistry (IHC) results (Fig. 7b) demonstrated that the expression of extracellular matrix components in the nucleus pulposus (NP), specifically aggrecan (ACAN) and type II collagen (COL2), was significantly higher in the MnGAHs group compared to the other groups. Additionally, the expression of matrix metalloproteinase 13 (MMP13) was found to be the lowest in the MnGAHs group. Meanwhile, the expression levels of p65, p53 and p21 in MnGAHs group were significantly lower than those in NS group (Fig. 8a). In the MnGAHS group, the expression of CD86 decreased while the expression of CD206 increased, indicating that the polarization of macrophages towards M2 macrophages was conducive to alleviating inflammation (Fig. 8b). These findings suggest that MnGAHs treatment significantly alleviated puncture-induced IVDD in the Sprague-Dawley rat model.

## 3 Conclusion

In conclusion, this study presents a novel approach for treating IVDD through the development of injectable Mn^2+^/glycyrrhizic acid hydrogels. By exploiting the ability of Mn ions to form salt bridges with GA molecules, we successfully synthesized a hydrogel that mimics the antioxidant activities of both SOD and CAT. Our findings indicate that MnGAHs can reduce oxidative stress and prevent the senescence of NPMSC, thereby mitigating cell aging and oxidative stress and enhancing their regenerative potential. Additionally, MnGAHs promote the polarization of macrophages toward the anti-inflammatory M2 phenotype, which helps to alleviate the inflammatory microenvironment associated with IVDD. The ROS-p53-p21 axis is identified as a key mechanism underlying the therapeutic effects of MnGAHs in reversing IVDD confirmed by integrating theoretical calculations with public database analyses and Western blot. In vivo rat model of IVDD also demonstrated that MnGAHs significantly improved IVDD progression. Taken together, this work offers novel insights for future treatments of IVDD by targeting the underlying pathophysiological processes including cell oxidative stress and inflammation.

## CRediT authorship contribution statement

**Yudong Fu:** Writing – review & editing, Writing – original draft, Investigation, Funding acquisition, Data curation, Conceptualization. **Hua Sun:** Writing – review & editing, Writing – original draft, Methodology, Formal analysis, Data curation, Conceptualization. **Yongchao Jin:** Software, Methodology, Investigation, Formal analysis, Conceptualization. **Shaohui Cheng:** Visualization, Resources, Conceptualization. **Yanyi Wu:** Visualization, Software, Conceptualization. **Chen Liu:** Writing – original draft, Data curation, Conceptualization. **Lei Fan:** Resources, Investigation, Conceptualization. **Juqun Xi:** Supervision, Funding acquisition, Conceptualization. **Shixin Li:** Visualization, Supervision. **Liang Zhang:** Visualization, Supervision, Funding acquisition, Conceptualization.

## Ethics approval statement

All procedures involving animals were approved by the Experimental animal Ethics Committee of Yangzhou University (No. 202403237).

## Consent for publication

All authors consent to the publication of the article.

## Funding

This work was supported by grants from the 10.13039/100014717National Natural Science Foundation of China (Grant No. 82172462), the 10.13039/501100004608Natural Science Foundation of Jiangsu Province (No. BK20231329), the Jiangsu Province sixth phase “333 Project” (No. 11), the Science and Innovation Fund for Yangzhou University students (No. XCX20230816) and Cross Cooperation Special Project of Northern Jiangsu People's Hospital (No. SBJC21014).

## Declaration of competing interest

The authors declare that they have no known competing financial interests or personal relationships that could have appeared to influence the work reported in this paper.

## Data Availability

All data in this study are available from the corresponding author upon reasonable request. No datasets were generated or analyzed during the current study.
