# Supplementary material for: Self-assembled antioxidant enzyme-mimicking hydrogel: Targeting oxidative stress and macrophage organization for improving degenerated intervertebral discs
Source: Mater Today Bio. 2025 Feb 19;31:101586. doi: 10.1016/j.mtbio.2025.101586 (PMC11923825; doi:10.1016/j.mtbio.2025.101586)
Supplement: Multimedia component 1 [file mmc1.docx]

**Supporting information**

**Self-assembled Antioxidant Enzyme-mimicking Hydrogel: Targeting Oxidative Stress and Macrophage Organization for Improving Degenerated Intervertebral Discs**

Yudong Fu^1†^, Hua Sun^1,2†^, Yongchao Jin^3^, Shaohui Cheng^4^, Yanyi Wu^1^, Chen Liu^1,2^, Lei Fan^4^, Juqun Xi^1,5*^, Shixin Li^3*^, Liang Zhang^2*^

^1^Institute of Translational Medicine，School of Medicine，Yangzhou University，Yangzhou，Jiangsu 225001，PR China

^2^Department of Orthopedics, Northern Jiangsu People’s Hospital Affiliated to Yangzhou University, Yangzhou, Jiangsu 225001, PR China

^3^College Of Bioscience and Biotechnology, Yangzhou University, Yangzhou, Jiangsu 225009, China

^4^School of Chemistry and Chemical Engineering, Yangzhou University, Yangzhou, Jiangsu 225002, PR China

^5^The Key Laboratory of the Jiangsu Higher Education Institutions for Integrated Traditional Chinese and Western Medicine in Senile Diseases Control (Yangzhou University)，Yangzhou，Jiangsu 225001，PR China

^†^Yudong Fu and Hua Sun contributed equally to this work.

^*^Correspondence:

Liang Zhang, Email: zhangliang6320@sina.com

Shixin Li, Email: lisx@yzu.edu.cn

Juqun Xi, Email: xijq@yzu.edu.cn

**Materials and methods**

**Synthesis of MnGAHs**

GA and MnCl_2_·4H_2_O were dissolved in water, respectively. Then, the MnCl_2_·4H_2_O aqueous solution and the GA aqueous solution are simply mixed according to the same volume ratio. Their final concentrations were 5mg/mL (GA) and 1mg/mL (MnCl_2_·4H_2_O) respectively. Finally, the gelation of MnGAHs was evaluated by tube inversion method, that is, no visual flow was observed after hydrogel formation.

**SEM, FTIR and XPS experiments**

MnGAHs fixed GA at 5.0 mg/mL and Mn^2+^ at 1.0 mg/mL was tested on Scanning electron microscope (S-4800II, Hitachi, Japan), Transform infrared spectroscopy (FTIR) was carried out using an FTIR-8300 series spectrometer (Shimadzu, Japan) and X-ray photoelectron spectroscopy (XPS) (ESCALAB 250Xi, Thermo Scientific, USA).

**Determination of binding affinity by isothermal titration calorimetry (ITC)**

GA (10 μM) in ddH_2_O were added to the sample cell. Then the MnCl_2_ solution (1 mM) in ddH_2_O were injected into the sample cell at a controlled rate. As the GA and MnCl_2_ molecules interact, heat is absorbed causing a change in temperature in the sample cell. A reference cell was prepared serving as a reference for background heat. The temperature difference between sample and reference cells were monitored during the titration.

**Rheological analysis of MnGAHs**

For rheological evaluation, MnGAHs fixed GA at 5.0 mg/mL and Mn^2+^ at 1.0 mg/mL was tested on a rotating rheometer (RS600, Thervno, USA). The frequency scanning, strain scanning and time scanning were carried out respectively. The hydrogel was scanned at alternating strain of 1% and 100%, and the strain interval was 60s.

**Swelling experiment and degradation experiment of MnGAHs**

**Swelling experiment**. Dry MnGAHs was prepared and then soaked in PBS (pH = 6.5) at 37 ℃. Remove the hydrogel at different points in time and weigh it. Finally, the swelling ratio is calculated.

**Degradation experiment.** MnGAHs was first prepared, then soaked in PBS (pH = 6.5) and placed in a constant temperature shaker (37 ℃, 50 rpm). Remove the hydrogel at different points in time and weigh it. Finally, the degradation ratio was calculated.

**Oxidative stress index detection**

The levels of hydrogen peroxide (H_2_O_2_), superoxide dismutase (SOD) and malonaldehyde (MDA) were measured by H_2_O_2_ Colorimetric Assay Kit (Elabscience), SOD assay kit (Beyotime) and MDA assay kit (Beyotime) as per the operations of supplier.

**Simulation details**

***Assembly of GA molecules and Mn^2+^.*** To analyze assembly structure, molecular dynamics simulation was performed using Gromacs 2020.6.^[1]^ In the simulation system, GA molecules and Mn^2+^ ions were randomly inserted into the simulation box, which was subsequently filled with the water molecules. The CHARMM36 all-atom force field was employed to parameterize the molecules, while TIP3P model was used for water molecules. After energy minimum and quick equilibrium with constant bead number, volume, and temperature (NVT) of 1 ns, the systems were equilibrated for 200 ns under constant bead number, pressure, and temperature (NPT) to achieve stable assembly structures. In the simulation, short-range interactions were truncated at 1.2 nm, and long-range Coulomb interactions were computed using the particle-mesh Ewald summation method.^[2]^ Pressure was maintained at 1 bar using the isotropic Parrinello-Rahman barostat with a coupling constant of 4 ps and a compressibility of 4.5 × 10^−5^ bar^−1^ in all three directions. The temperature was maintained at 298.15 K using the V-rescale thermostat with a coupling constant of 0.1 ps. The Lennard-Jones (LJ) potential was smoothly shifted to zero between 0.9 nm and 1.2 nm to minimize cutoff noise. Periodic boundary conditions were applied in all three directions. The time step was 2 fs, with the neighbor list was updated every 10 steps. Snapshots were visualized using PyMOL.^[3]^

***Binding modes of*** ***GA molecule with various proteins.*** We combined molecular dockding and molecular dynamcis simulations to analyze the binding modes of the GA molecule with various proteins, including p65, p53 and p21. All molecular docking simulations were performed using the AutoDock Vina (version 1.1.2),^[4]^ with the ligand search space encompassing the entire protein surface based on the crystal structures. To assess the stability of the resulting complexes, molecular dynamics simulations were performed using the CHARMM36 all-atom force field and GROMACS 2020.6.^[1]^ The docking results provided the initial structures for these simulations. Each complex was centered in a cubic box and solvated with the TIP3P water model. After initial energy minimization and 1 ns NVT equilibration, each system was subjected to a 50 ns NPT simulation to confirm the stability. The molecular dynamics parameters used in these simulations were the same as that in the GA and Mn²⁺ assembly simulations.

**Network pharmacology**

We used the Swiss Target Predictions (http://www.swisstargetprediction.ch/), Drugbank (http://www.drugbank.ca) to obtain GA-related targets. We integrated and deleted the GA duplicate genes obtained from the above two databases and determined the remaining as the final GA target genes. We got IVDD-related targets by GeneCards (https://www.genecards.org/) and DisGeNET (https://www.disgenet.org/). We obtained GA and IVDD common targets by the jven (https://jvenn.toulouse.inra.fr/app/index.html). The STRING (https://www.string-db.org) database was used for protein-protein interaction (PPI) analysis of the targets. The DAVID (https://david.ncifcrf.gov) database was employed for the enrichment of gene ontology (GO) functional enrichment analysis and the Kyoto Encyclopedia of Genes and Genomics (KEGG) pathway analysis. Through these analyses, the potential pathway and key therapeutic targets of GA in the treatment of IVDD were obtained. Subsequently, the network analysis and visualization were constructed by Cytoscape 3.10.0 software.

**Cell hydrogel treatment methodology**

We first spread MnGAHs on the bottom of the cell pore plate and then planted the cells on MnGAHs for culture to carry out follow-up research.

**Cytocompatibility evaluation in vitro**

The NPMSC used in this paper were isolated from the tail IVD of SD rats. Healthy SD rats (200-220 g, 2-3 months old) were sacrificed using an overdose of sodium pentobarbital. IVD tissues were carefully collected from the caudal discs and digested with 0.2% (w/v) type II collagenase in an incubator at 37℃ for 4 h. The isolated cells in supernatant were cultured in DMEM (KeyGEN) medium supplemented with 10% (v/v) fetal bovine serum (FBS, KeyGEN) and 1% penicillin–streptomycin (Invitrogen), and maintained in cell incubator under a 5% CO_2_ atmosphere at 37℃. The culture medium was changed every two days, and cells were removed for subsequent experiments after the third passage. The cytotoxicity in vitro of MnGAHs in rat NPMSC was evaluated by staining with MTT cell survival assay. Specifically, 5000 cells were plated in a 96 well plates and cultured in DMEM (KeyGEN) medium supplemented with 10% (v/v) fetal bovine serum (FBS, KeyGEN) and 1% penicillin–streptomycin (Invitrogen), and maintained in cell incubator under a 5% CO_2_ atmosphere at 37℃. To determine cell viability, the cells were incubated for 1 days, respectively, with culture medium containing GA, MnCl_2_ and MnGAHs. In the MnGAHs group, cells were cultured on MnGAHs. After 24 h, cell viability was measured using a microplate reader (SPARK 10 M) at an excitation wavelength of 570 nm.

**Hydrogen peroxide-induced oxidative stress methodology**

We implanted the cells into the cell pore plate and, after 24 h, added 200 µM H_2_O_2_ to the cells that needed hydrogen peroxide to induce oxidative stress. Perform other operations two hours later.

**MTT cell survival assay**

5000 NPMSCs were plated in a 96 well plates and cultured in DMEM (KeyGEN) medium supplemented with 10% (v/v) fetal bovine serum (FBS, KeyGEN) and 1% penicillin–streptomycin (Invitrogen), and maintained in cell incubator under a 5% CO_2_ atmosphere at 37℃. After 24 h, 200 µM H_2_O_2_ was added to each group except Control group. After 2 h, GA was added to H_2_O_2_ + GA group, MnCl_2_ was added to H_2_O_2_ + MnCl_2_ group, and cells of H_2_O_2_ + MnGAHs group were cultured on MnGAHs. After 24 h, cell viability was measured using a microplate reader (SPARK 10 M) at an excitation wavelength of 570 nm by MTT assay.

**Cell cycle assay**

The cells of logarithmic growth phase were collected and inoculated with appropriate culture medium‌. After the corresponding time of drug treatment and culture, the medium was removed, pancreatic enzyme was used to digest and the cells were collected by centrifugation. The cells were then cleaned with PBS twice and appropriate amount of PBS was added to resuspend cells after the residual PBS was removed. Under dark condition, the cells were added with RNase to digest RNA, then propyl iodide (‌PI) was added for staining, and incubated for 30 min at room temperature. The treated cells were transferred into a flow tube for detection using flow cytometry to determine the proportion and distribution of the phases of the cell cycle.

**Flow cytometry**

After NPMSC are treated with 200 µM H_2_O_2_, GA, MnCl_2_ and MnGAHs are treated separately. 1×10^6^ cells were collected, and Annexin V-FITC/PI apoptosis kit (KeyGEN) was used to perform flow cytometry analysis and quantitative analysis of these cells using FACS Aria Fusion (BD) according to the experimental method.

**Beta galactosidase staining**

NPMSC were seeded into 6-well plate. When cell adhesion and fusion reached more than 80%, H_2_O_2_ (200 µM) was added to the 6-well plate for 24 h. Subsequently, The GA group was given GA treatment. The MnCl_2_ group was given MnCl_2_ treatment. The MnGAHs group was given MnGAHs treatment. The control group was not given H_2_O_2_ treatment. Senescence-Associated β-Galactosidase (SA-β-Gal) Stain Kit (Solarbio) was used to stain senescent cells. The positive rate of cells was analyzed by ImageJ.

**Western blot analysis**

Cells were lysed with RIPA buffer (Sigma‐Aldrich) to obtain total proteins, and a BCA Kit (Thermo) was utilized for the quantification of these protein contents. Proteins were separated by sodium dodecyl sulphate‐polyacrylamide gel electrophoresis and then were transferred to PVDF membranes (All from Beyotime). After the overnight cultivation with specific primary antibodies at 4°C, the membranes were incubated with secondary antibodies (1:500, Proteintech). The protein bands were visualized with enhanced chemiluminescence reagents and then analyzed with the Tanon5200 system. The following were the primary antibodies: tumor protein p53 (p53, 1:1000, Proteintech), cyclin-dependent kinase inhibitor 1A (p21, 1:1000, Proteintech), v-rel reticuloendotheliosis viral oncogene homolog A (NF-κb p65, 1:1000, Proteintech) and GAPDH (1:1000, Proteintech). The results were analyzed using ImageJ.

**Immunofluorescence anlysis**

Immunofluorescence staining was conducted as previously described to assess CD86 and CD206 expression in the RAW264.7 cells. In immunofluorescence staining of M1 macrophages, we first implanted RAW264.7 cells onto the cell crawl. After 24h, LPS (1 µg/mL) and INF-γ (10 ng/mL) were added to LPS + INF-γ group, LPS + INF-γ + GA group, LPS + INF-γ + MnCl_2_ group, and LPS + INF-γ + MnGAHs group. 2 h later, GA was added to the LPS + INF-γ + GA group, MnCl_2_ was added to the LPS + INF-γ + MnCl_2_ group, and the cell slides were cultured on MnGAHs in the LPS + INF-γ + MnGAHs group. In immunofluorescence staining of M2 macrophages, we first implanted RAW264.7 cells onto the cell crawl. After 24 hours, LPS (1 µg/mL) and IL-4 (10 ng/mL) were added to LPS + IL-4 group and LPS (1 µg/mL) was added to LPS + GA group, LPS + MnCl_2_ group and LPS + MnGAHs group. 2 h later, GA was added to the LPS + GA group, MnCl_2_ was added to the LPS + MnCl_2_ group, and the cell slides were cultured on MnGAHs in the LPS + MnGAHs group. After 24 h, the cells were washed twice with phosphate-buffered saline (PBS) and subsequently fixed using 4% paraformaldehyde for 10 min. The fixed cells underwent treatment with 0.1% Triton X-100 for 15 min, followed by incubation with anti-CD86 (1:1000, Proteintech) and anti-CD206 polyclonal antibody (1:1000, Proteintech) at 4℃ overnight. On the subsequent day, Red and green fluorescent secondary antibody (1:1000, Proteintech) were employed to label CD86 and CD206 antibodies, respectively, at room temperature for 1 hour. The cell nuclei were stained with bisBenzimide H 33258 (Hoechst, Beyotime). Fluorescent images were acquired using a Ultra-high resolution confocal laser microscopy (LEICA).

**Enzyme‐linked immunosorbent (ELISA) experiments**

We first planted RAW onto a 6-well plate, and after 24 hours, LPS (1 µg/mL) was added to all groups except the control group. Two hours later, GA was added to LPS + GA group and MnCl_2_ was added to LPS + MnCl_2_ group, and the cells of LPS + MnGAHs group were cultured on MnGAHs. After treatment for 24h, the cell supernatant was extracted for follow-up study. The supernatant levels of tumor necrosis factor‐α (TNF‐α), interleukin‐1beta (IL‐1β) and interleukin‐10 (IL‐10) were measured using quantitative ELISA kits (YIFEIXUE) in line with the producer's instructions.

**Intracellular ROS scavenging ability evaluation**

The antioxidant capacity of MnGAHs in vitro was characterized by reactive oxygen species detection kit (Beyotime Biotechnology). Briefly, NPMSC were seeded into 6-well plate. When cell adhesion and fusion reached more than 80%, H_2_O_2_ (200 µM) was added to the 6-well plate for 24 h. Subsequently, The GA group was given GA treatment. The MnCl_2_ group was given MnCl_2_ treatment. The MnGAHs group was given MnGAHs treatment. The control group was not given H_2_O_2_ treatment. After 1 day, 2′, 7′-dichlorofluorescein-diacetate (DCFH-DA) was diluted 1:1000 with serum-free medium to make the final concentration 10 µM, the cell culture medium of each group was removed, and the cells were washed twice with PBS, Subsequently, the diluted DCFH-DA solution was added, followed by incubation in the dark for 30 min. After incubation, the cells were washed twice with serum-free medium to remove excess DCFH-DA. Then, it was incubated with Hoechst 33342 at room temperature for 10 minutes and washed with serum-free medium 2 times. Finally, all samples were observed using an inverted fluorescence microscope. Fluorescence intensity was measured using Image J software.

**Transwell migration assay**

Migration of NPMSC was detected in the 6-well plate. Inoculated with 1×10^4^ NPMSC, the migration system was cultured at 37 ℃ and 5% CO_2_ for 24 h. Then, The GA group was given GA treatment. The MnCl_2_ group was given Mn^2+^ treatment. The MnGAHs group was given MnGAHs treatment. The control group was not given treatment. Finally, the NPMSC were observed using an inverted fluorescence microscope and imaged. Migrating cells were quantitatively analyzed using ImageJ.

**Live/Dead Viability**

A Calcein/PI Live/Dead Viability/Cytotoxicity Assay Kit (Beyotime) was used to detect the level of apoptosis following the recommendations of manufacturer. The level of apoptosis was determined by green and red fluorescence in the nucleus. Five randomly selected fields were observed under a fluorescence microscope (ZEISS).

**Hemolysis assay**

Mouse blood was collected and centrifuged at 4 ℃ and 3500 rpm for 5 min. The resulting erythrocytes were resuspended in PBS. Then, 500 µL of MnGAHs was mixed with 500 µL of erythrocytes in a tube and incubated at 37 ℃ for 1 h. A positive control (Triton X-100) and a negative control (PBS) were included in the experiment to calculate the degree of hemolysis. After incubation, all samples were centrifuged at 3500 rpm for 5 min, and the upper solution was collected to measure the absorbance at 542 nm using a microplate reader.

**Rat IVDD model and the implantation of materials**

This study was conducted in accordance with the guidelines approved by the Experimental Animal Ethics Committee of Yangzhou University (No. 202403237). For in vivo experimentation, 2-3 months old Sprague-Dawley male rats were randomly selected from the Animal Center of Yangzhou University. The rats were then anesthetized using isoflurane inhalation. The surgical procedure for inducing intervertebral disc degeneration (IVDD) in rat models followed previously established methods. In summary, a 21-gauge needle was percutaneously inserted into the center of the nucleus pulposus (NP) through the annulus fibrosus at the Co 5-6 or Co 6-7 level. The needle was then rotated 180^◦^ and held in place for 5 s, with locking forceps clamped at a distance of 5 mm from the needle to ensure consistent depth. The control group (n = 5) underwent only anesthesia and skin incision, with no NP damage inflicted. The IVDD rats (n = 25) were divided into five groups with the control group (n = 5) as the negative control, and NS (n = 5), GA (n = 5), MnCl_2_ (n = 5), and MnGAHs (n = 5) were respectively implanted or injected into the IVD at the second weeks after surgery. All animals completed the study as planned, and there were no deaths or removals from the study.

**Imaging validation**

At four- and eight-weeks post-operation, all rats were imaged using a Magnetic Resonance Imaging (MRI) of IVD detected to monitor the degeneration or regeneration of IVD. The detailed MRI Pfirrmann grading system was shown as Table S1.^[5]^

**Histological analysis**

At the 8 th week post-operation, all rats were euthanized. IVD samples were collected, fixed in 4% paraformaldehyde, decalcified, and then embedded for sectioning. Histopathological characteristics were assessed using hematoxylin and eosin (H&E) staining and Safranin O-fast green staining (SO/FG). Histological results for assessing the disc degeneration were qualified by histological scores based on the previous method. The detailed Histological Grading Scale Category Grade was shown as Table S3.^[6]^

**Immunohistochemistry analysis**

IVD paraffin sections underwent antigen retrieval by incubation in citrate buffer (pH 6.0) (Servicebio, G1202) and microwaving on high heat for 6-8 min. To prevent nonspecific protein binding, sections were blocked with 5% bovine serum albumin (BSA) at room temperature for 30 min. Subsequently, sections were incubated overnight at 4℃ with primary antibodies anti-COL2, anti-MMP13 and anti-ACAN (1:1000, Proteintech). Following this, sections were incubated with appropriate secondary antibodies for 1 h at ambient temperature. Finally, sections were dehydrated, sealed, and digitally scanned using a slide scanner.

**Serum biochemical tests**

Serum levels of glutamic pyruvic transaminase (ALT), glutamic oxaloacetic transaminase (AST), blood urea nitrogen (BUN) and creatinine (CREA) were measured using Aspartate, Alanine Aminotransferase, Blood Urea Nitrogen and Creatinine Assay Kits, according to the manufacturer’s instructions.

**Statistical analysis**

GraphPad Prism 8.0 software (GraphPad Software Inc.) was used for statistical analysis. All data were presented as the mean ± standard deviation. Unpaired Student's t test was utilized to demonstrate the statistical differences between two groups. Differences among multiple groups were analyzed with one‐way analysis of variance, followed by Tukey's post hoc test. A value of p less than 0.05 was regarded to be statistically significant. All experiments are statistically significant, that is, the sample size is greater than or equal to 3.


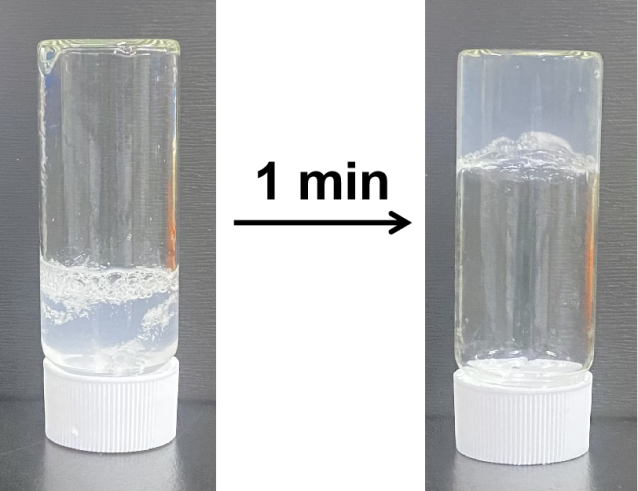


**Fig. S1** MnGAHs are formed within 1 min. GA: 5 mg/mL, MnCl_2_: 1 mg/mL.


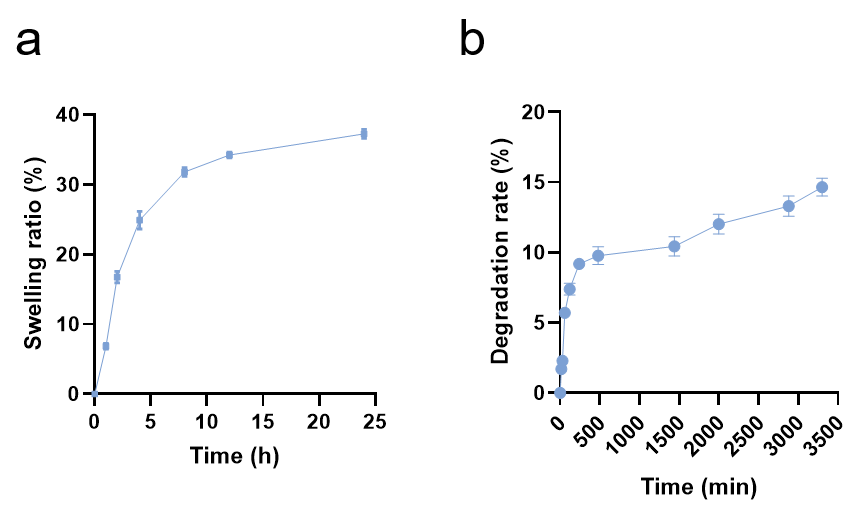


**Fig. S2** Results of swelling **(a)** and degradation **(b)** experiments of MnGAHs (n = 3).


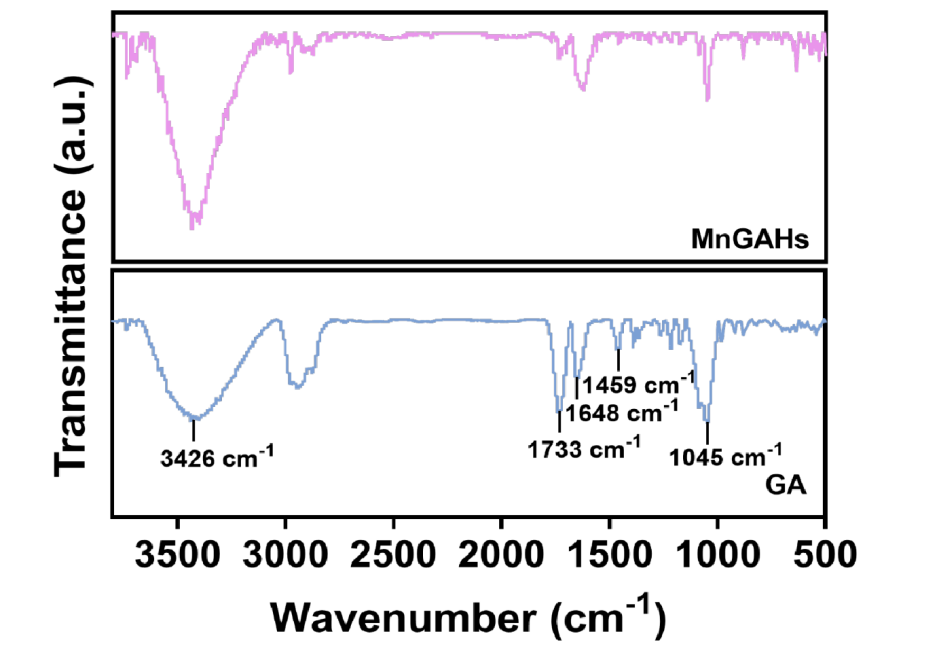


**Fig. S3** FT-IR spectra of pure GA and MnGAHs.


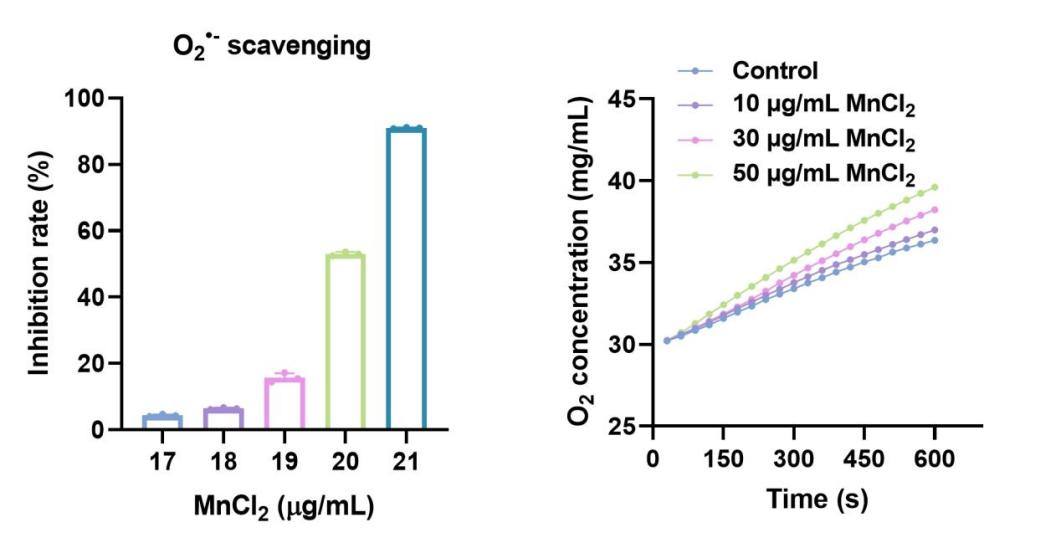


**Fig. S4** Antioxidant activity of MnCl_2_ under different concentrations (n = 3).


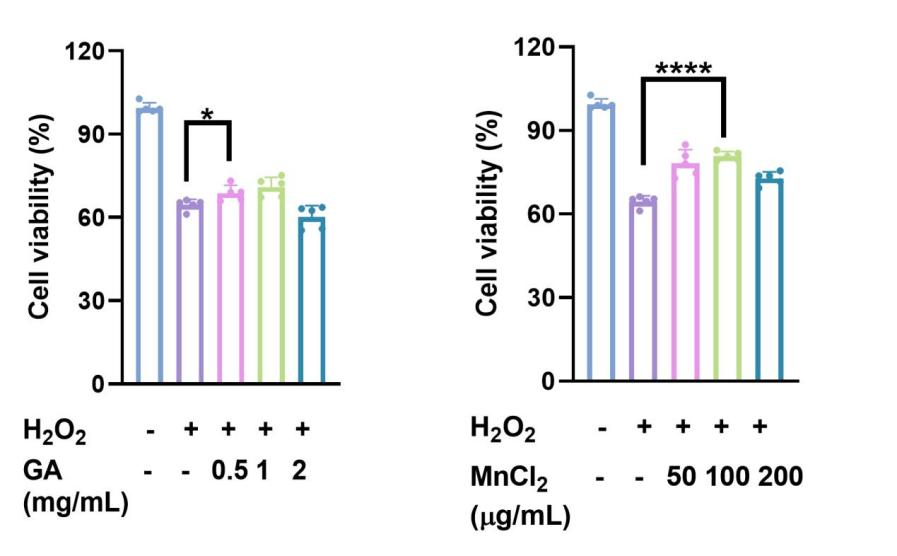


**Fig. S5** Cell viability of NPMSC treated with different concentrations of GA and MnCl_2_ (n = 5). H_2_O_2_: 200 µM *P< 0.05; ****P< 0.0001


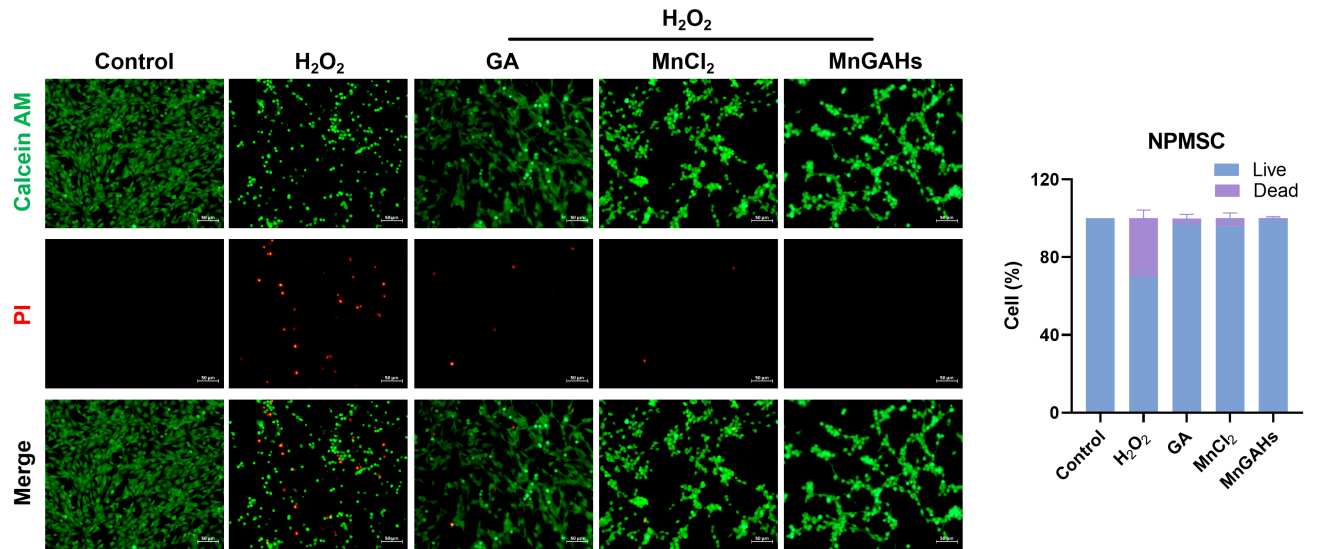


**Fig. S6** Calcein AM (live)/PI (dead) staining of NPMSC in difference groups (Scale bar = 50 µm, n = 3). H_2_O_2_: 200 µM, GA: 5 mg/mL, MnCl_2_: 1 mg/mL.


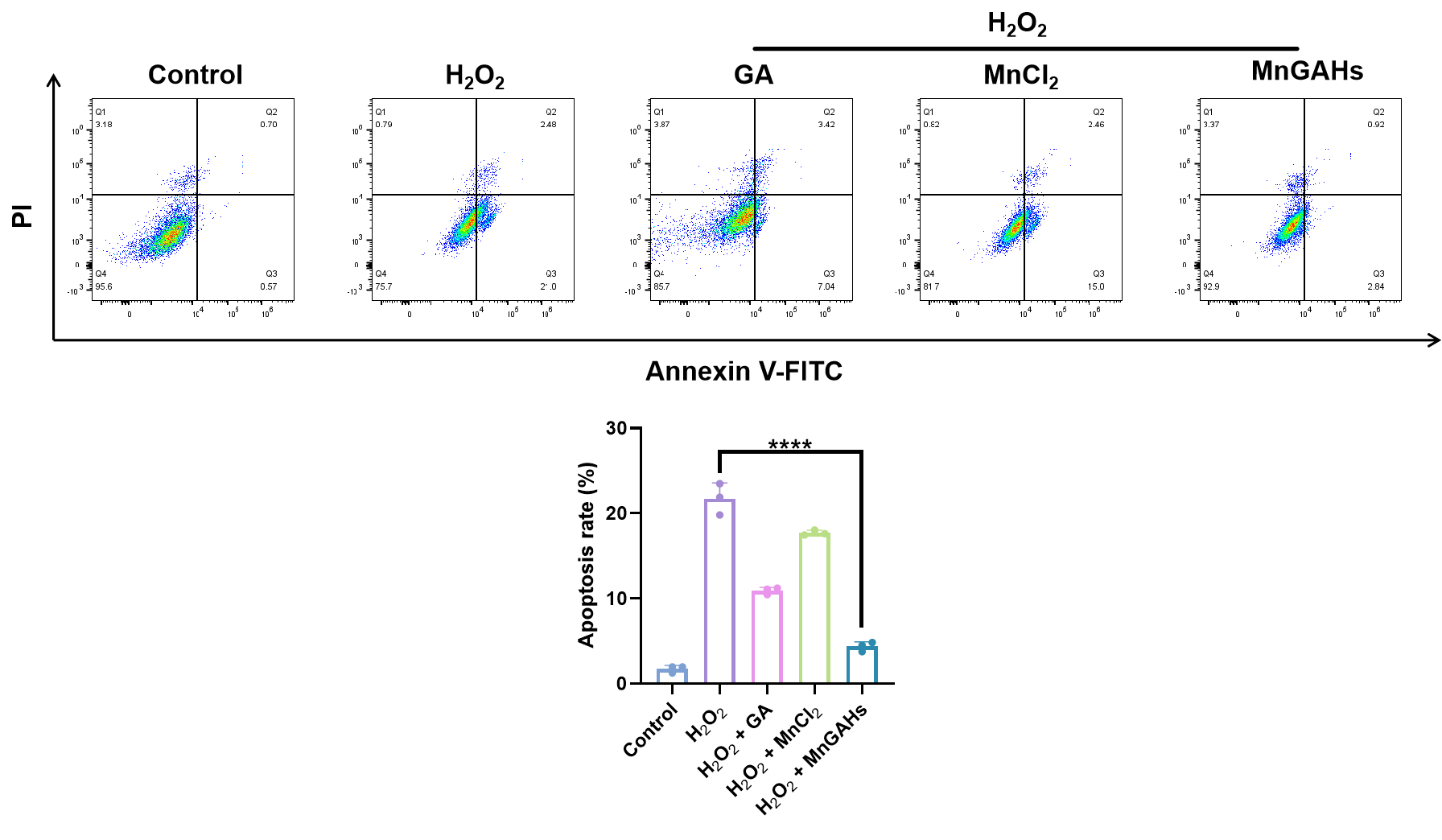


**Fig. S7** Flow cytometry results of apoptosis and corresponding quantitative analysis after different treatments (n = 3). ****P< 0.0001


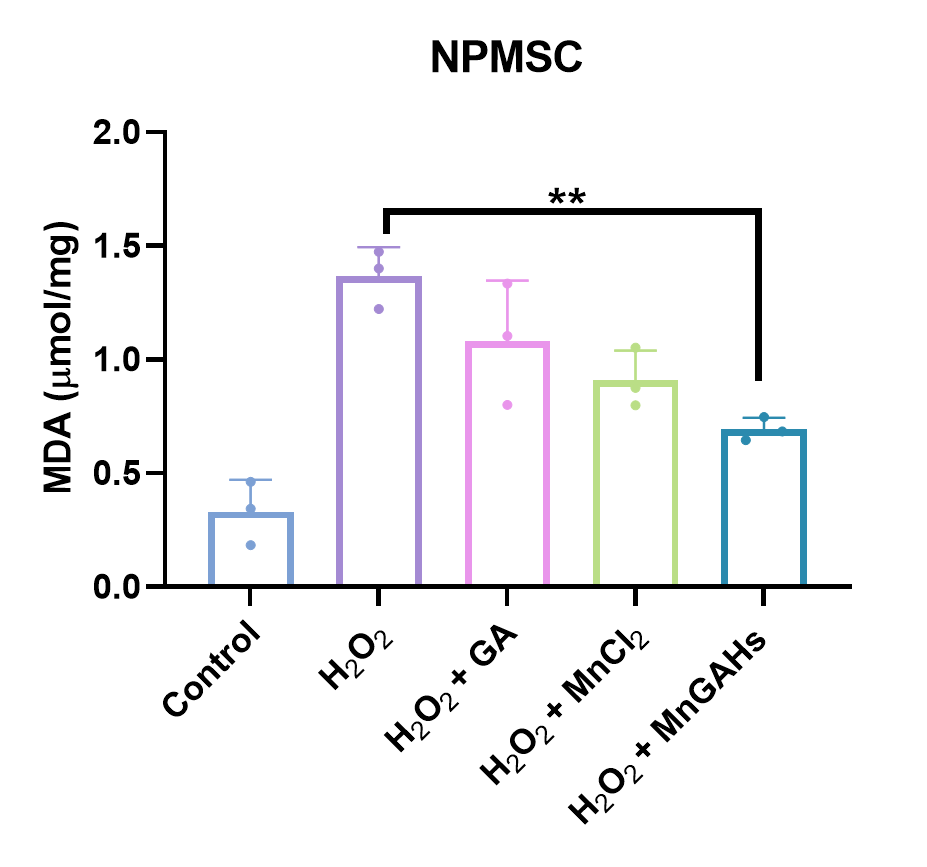


**Fig. S8** MDA concentration in NPMSC with different treatments (n = 3). H_2_O_2_: 200 µM, GA: 5 mg/mL, MnCl_2_: 1 mg/mL. **P< 0.01


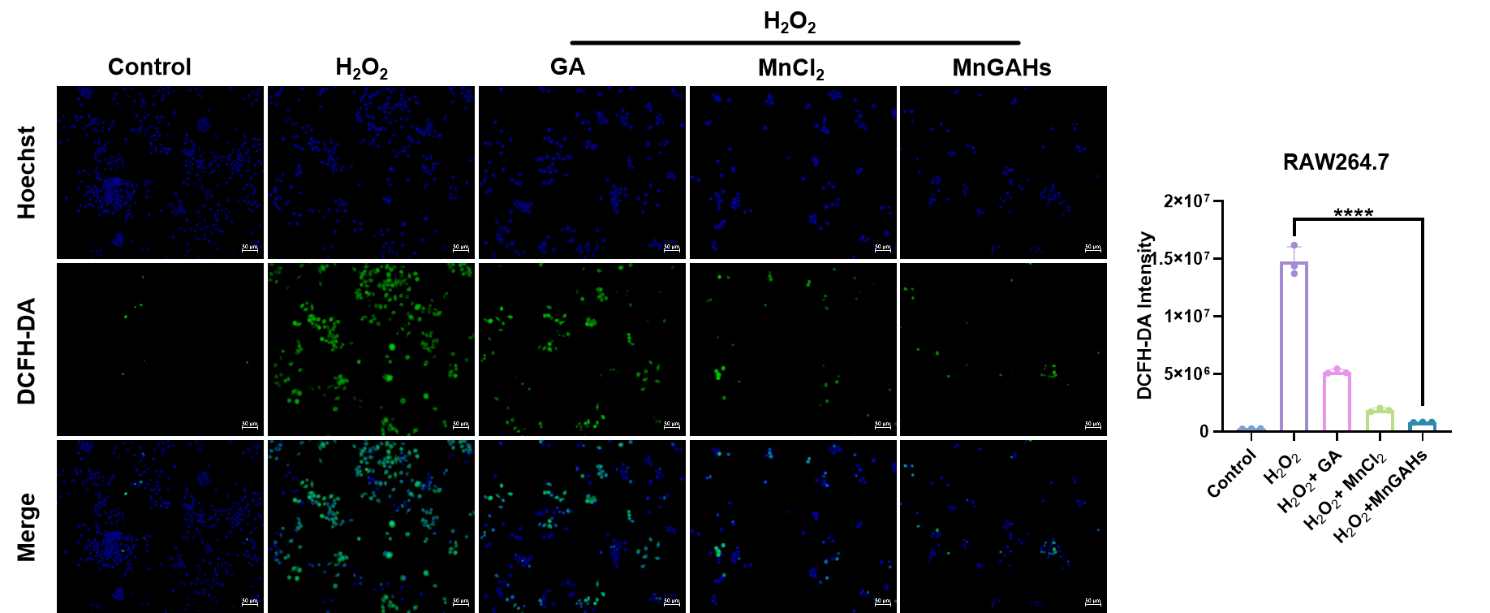


**Fig. S9** ROS levels (measured by the DCFH-DA assay) and corresponding statistical results in RAW264.7 cells after incubation with H_2_O_2_, followed by treatments with GA, MnCl_2_, and MnGAHs, respectively. The nucleus was stained with Hoechst. (Scale bar = 50 µm; n = 3). H_2_O_2_: 200 µM, GA: 5 mg/mL, MnCl_2_: 1 mg/mL. ****P< 0.0001


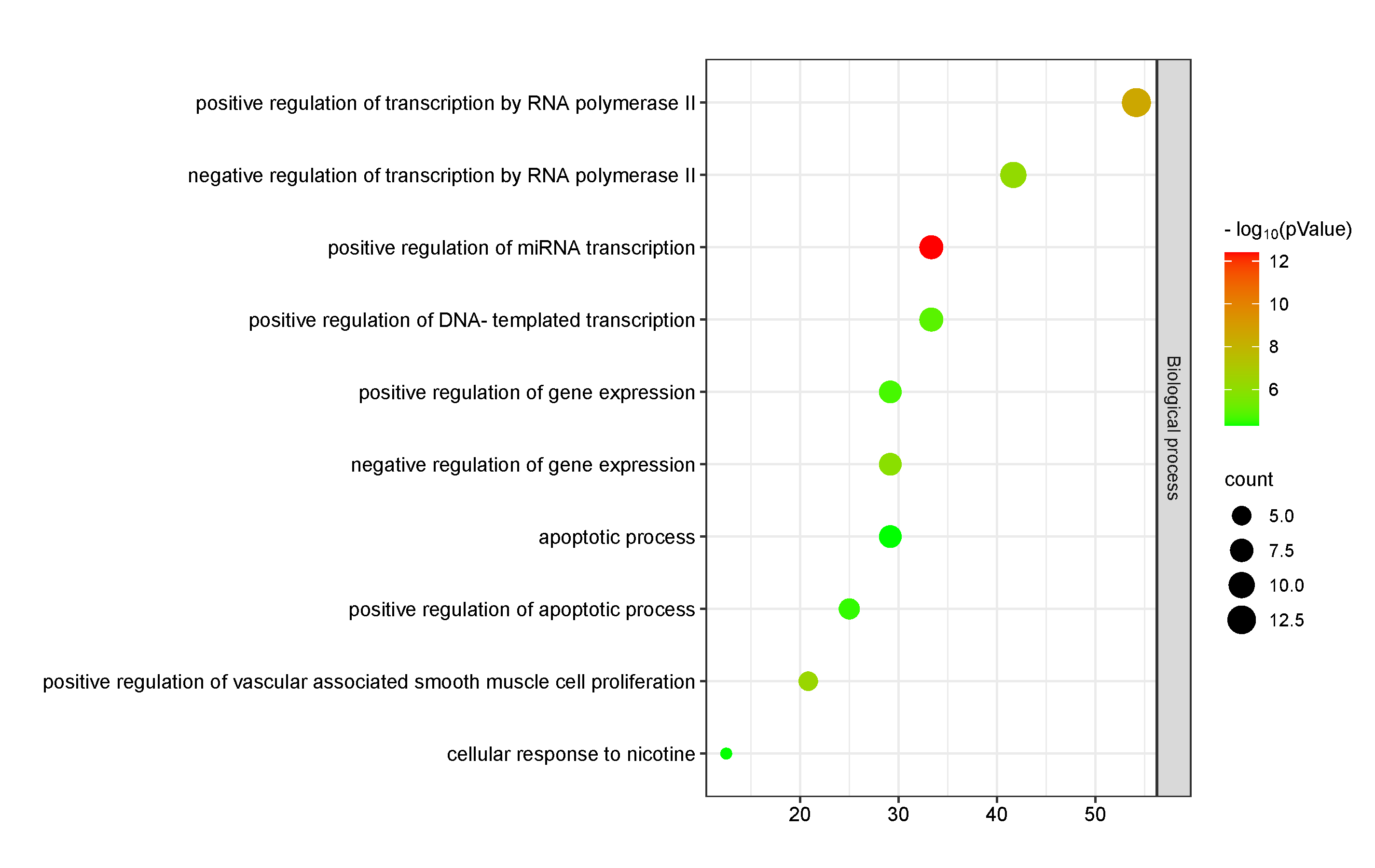

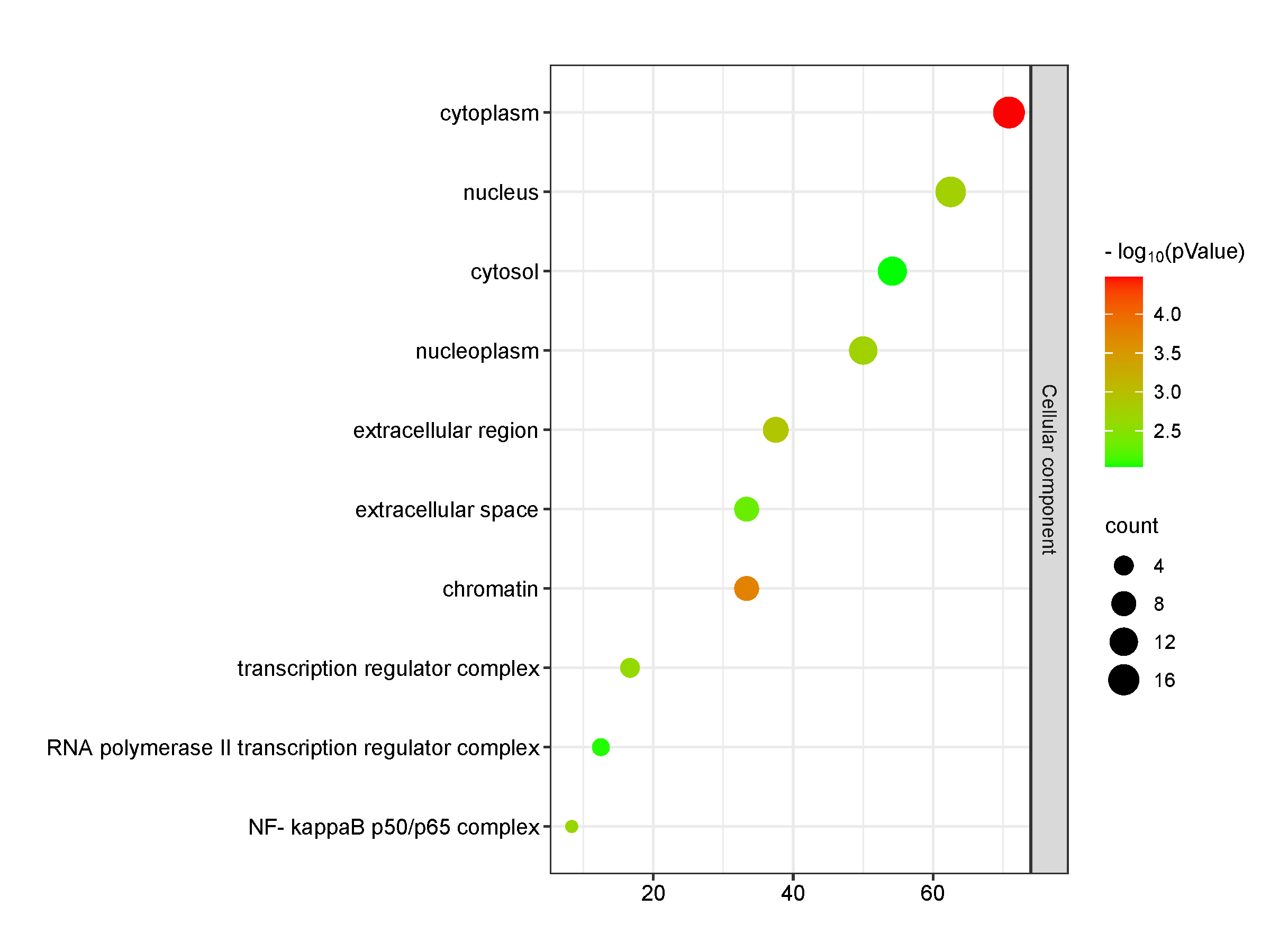

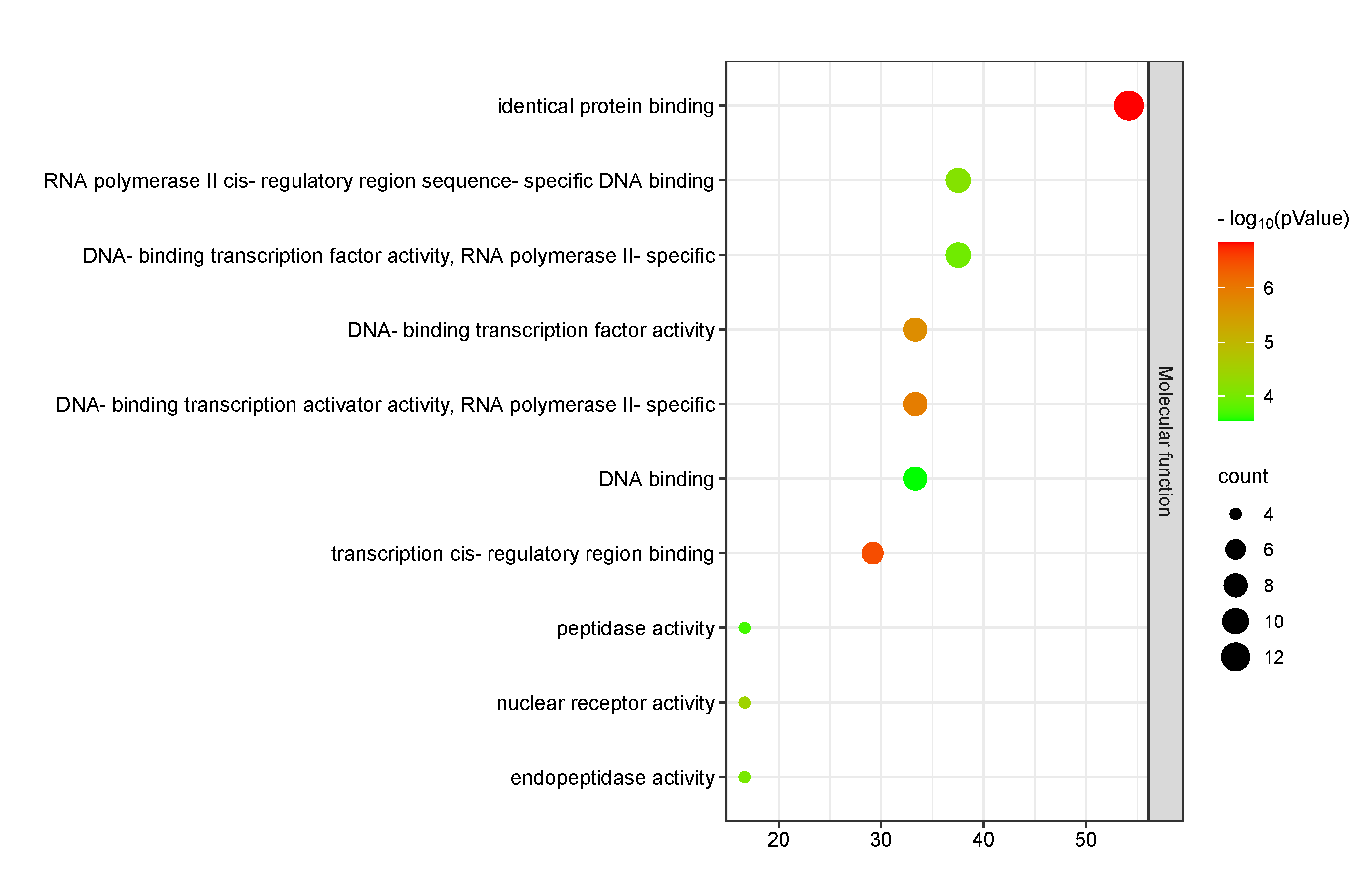


a

b

c

**Fig. S10** Bubble chart of the top 10 of the enriched. **(a)** biological processes, **(b)** cellular components and **(c)** molecular functions linked to the therapeutic effect of GA on IVDD.


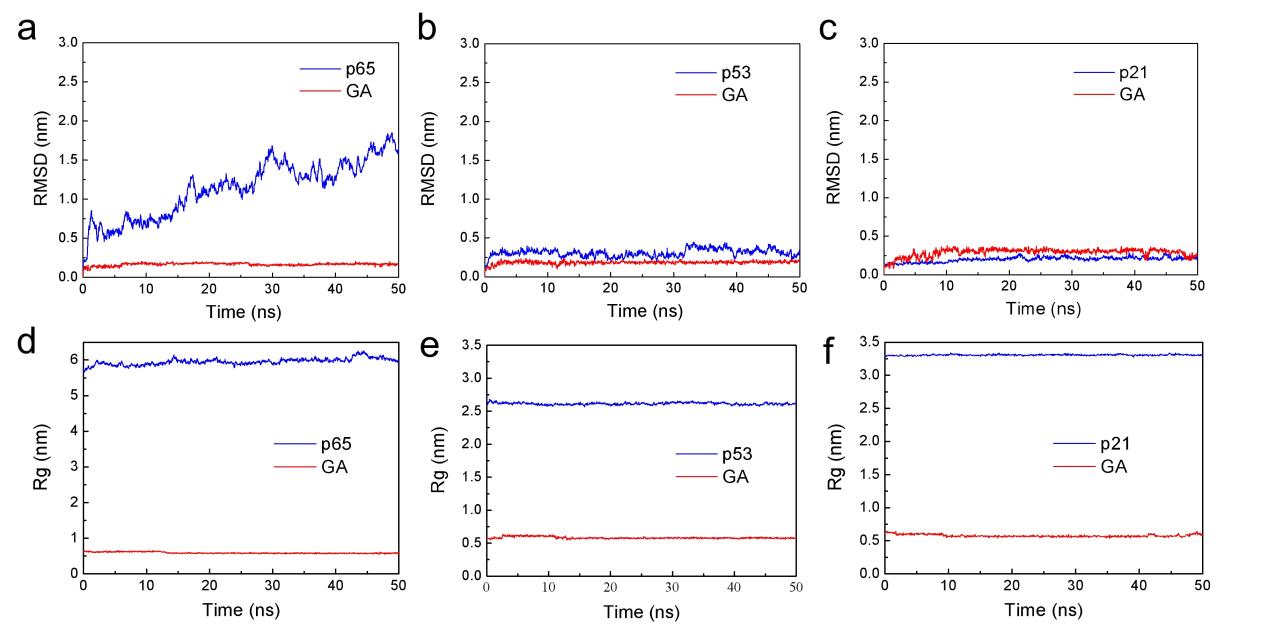


**Fig. S11** Time evolutions of root mean square displacement (RMSD) and gyration radius (Rg) for GA molecules and proteins.


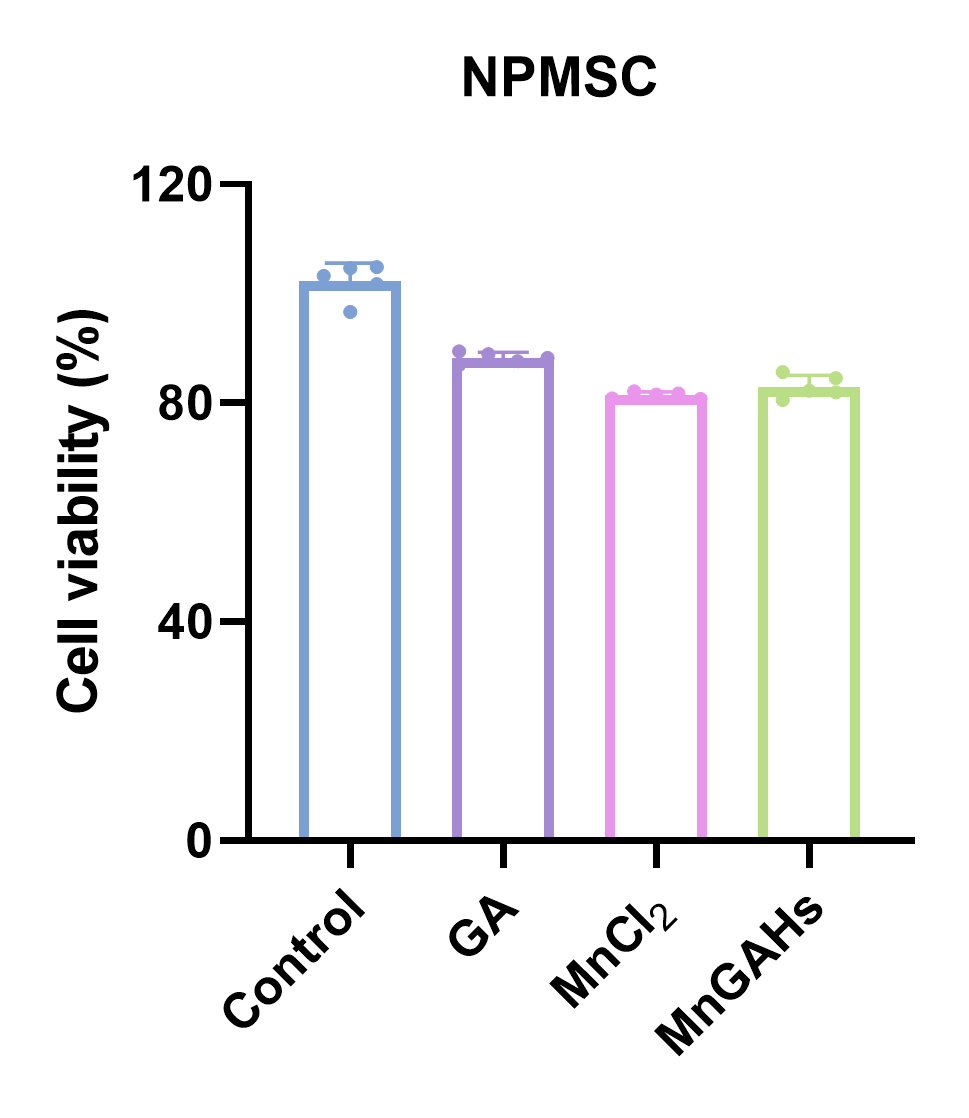


**Fig. S12** Cell viability of NPMSC under different treatments. GA: 5 mg/mL, MnCl_2_: 1 mg/mL (n = 5).


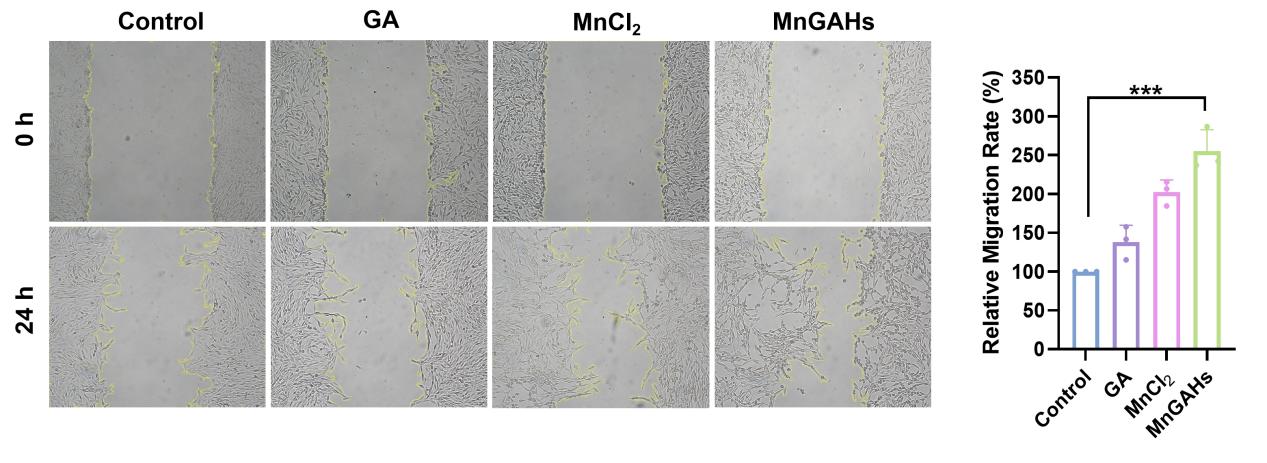


**Fig. S13** Migration map of NPMSC with different treatment and corresponding statistical analysis. GA: 5 mg/mL, MnCl_2_: 1 mg/mL (n = 3). ***P< 0.001


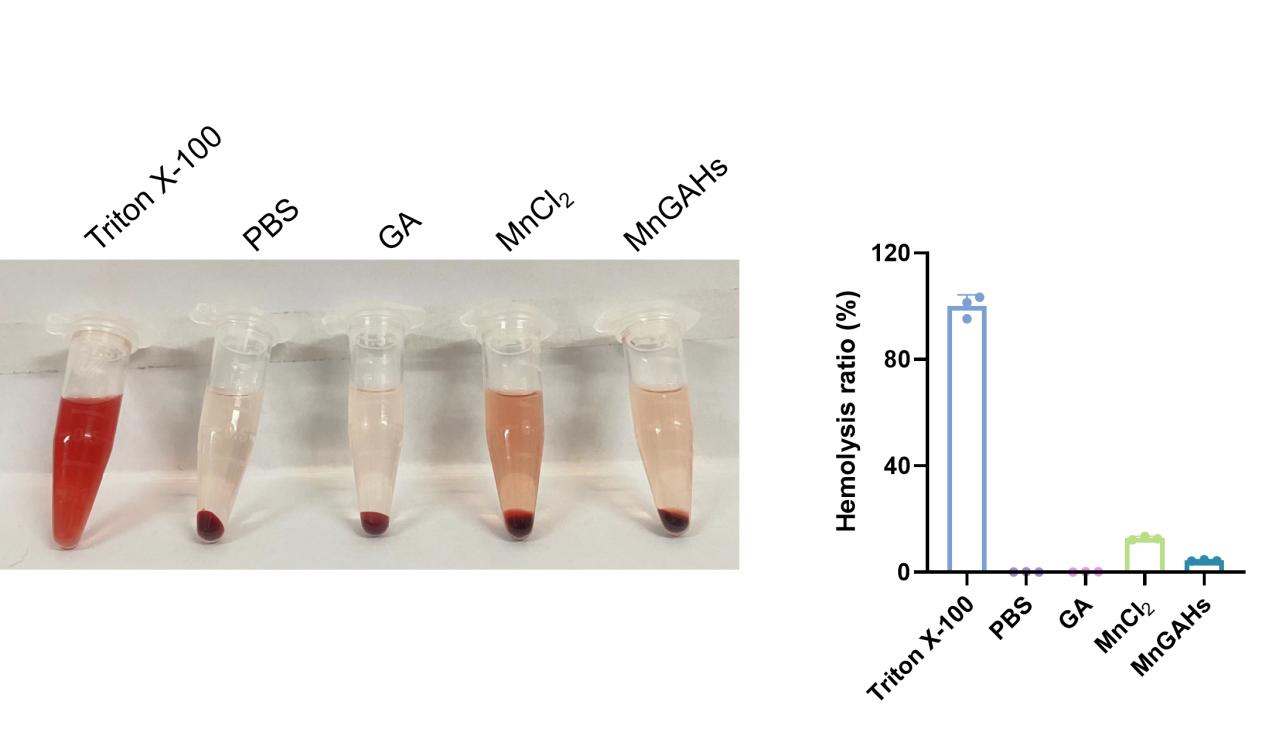


**Fig. S14** Percentage of hemolysis under different treatments. GA: 5 mg/mL, MnCl_2_: 1 mg/mL (n = 3).


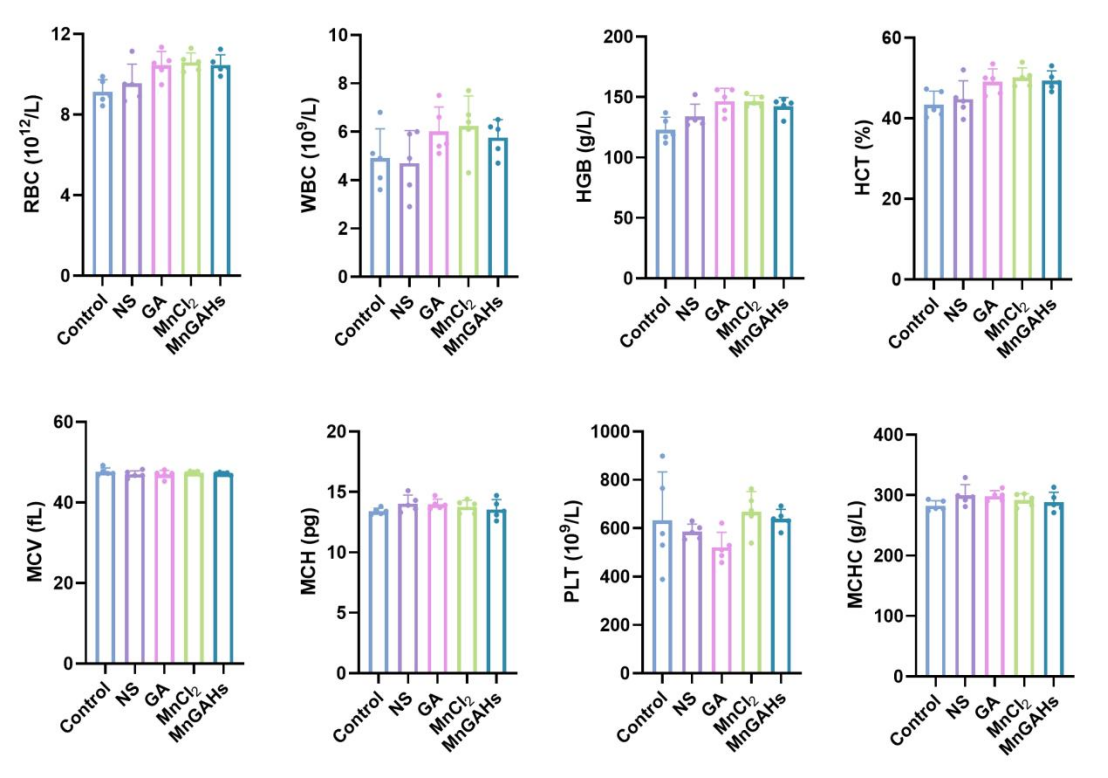


**Fig. S15** Standard hematological examination in different groups: red blood cells (RBC), white blood cells (RBC), hemoglobin (HGB), hematocrit (HCT), mean corpuscular volume (MCV), mean corpuscular hemoglobin (MCH), platelet (PLT) and mean corpuscular hemoglobin concentration (MCHC). GA: 5 mg/mL, MnCl_2_: 1 mg/mL (n = 5).


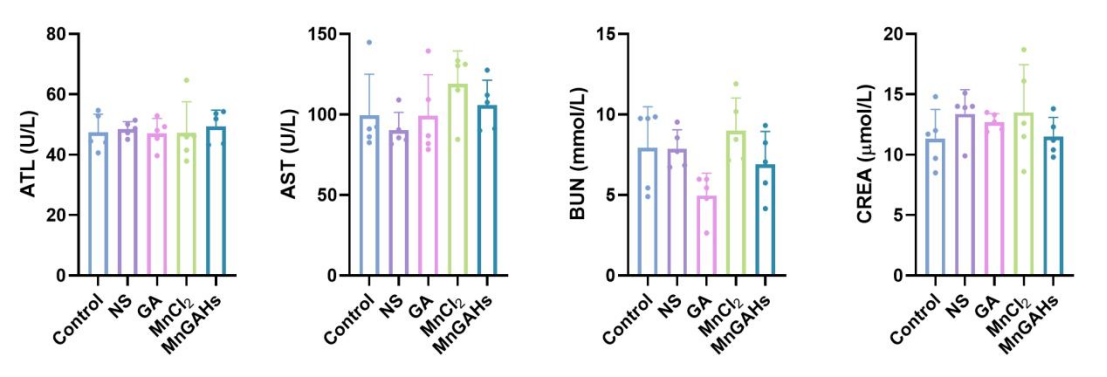


**Fig. S16** Biochemical indexes of each component: Serum levels of alanine transaminase (ALT), aspartate transaminase (AST), alkaline phosphatase (ALP), blood urea nitrogen (BUN), and creatinine (CERA). GA: 5 mg/mL, MnCl_2_: 1 mg/mL (n = 5).


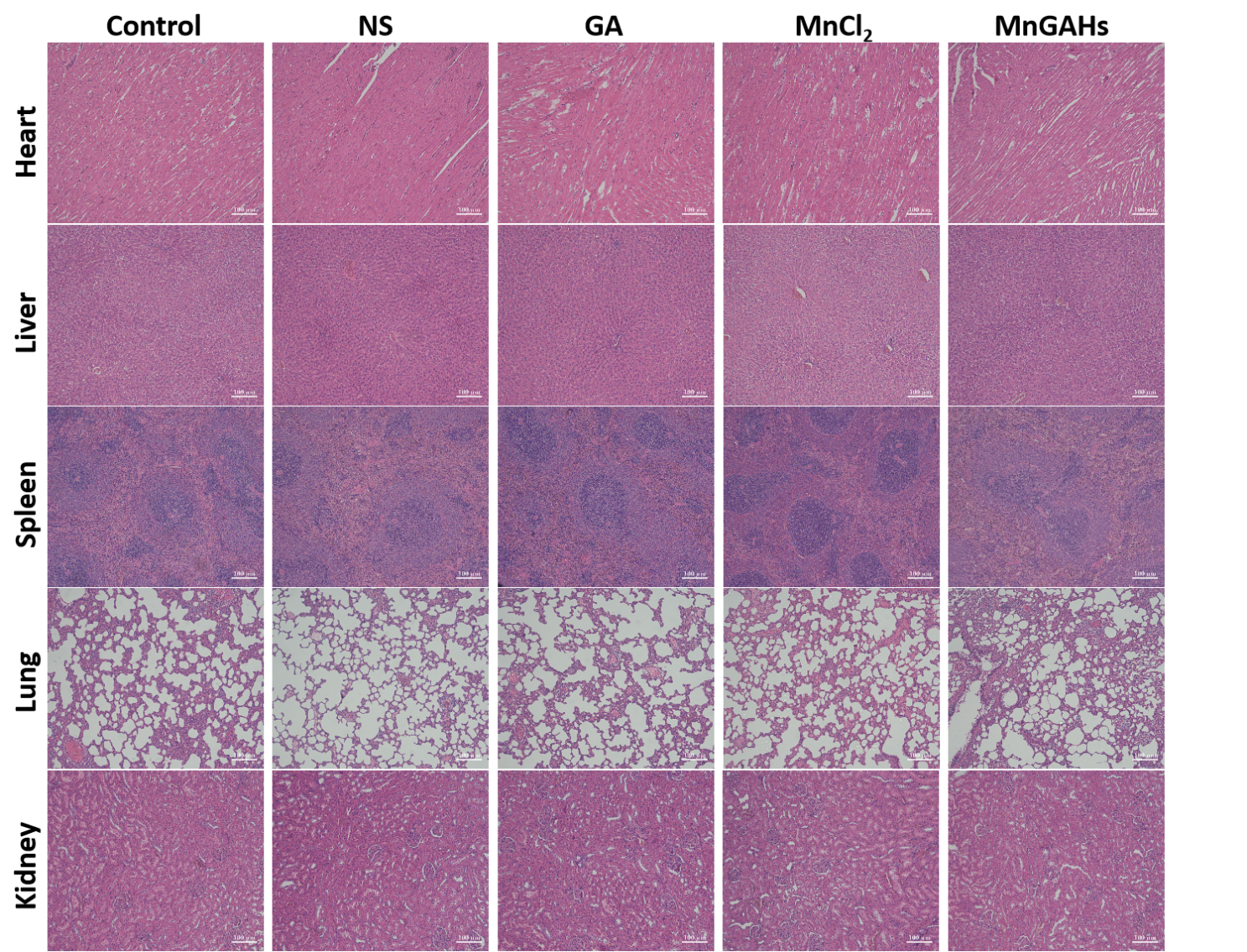


**Fig. S17** Histopathology images of dissected major organs (heart, liver, spleen, lung and kidney) stained with H&E of control and all experiment groups. GA: 5 mg/mL, MnCl_2_: 1 mg/mL (Scale bar = 100 μm).

**Table S1** MRI Pfirrmann grading system

**Table S2** MnGAHs formation at different GA and MnCl_2_ concentrations. (√: gelation; ×: No gelation).


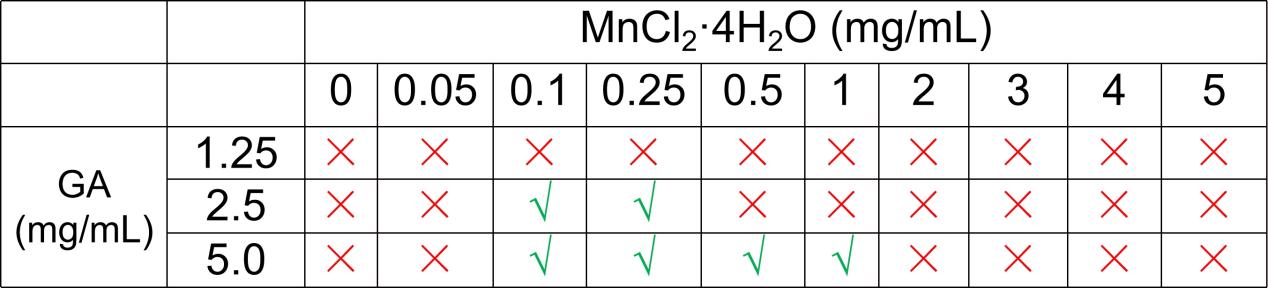


**Table S3** Definition of a histological grading scale category grade


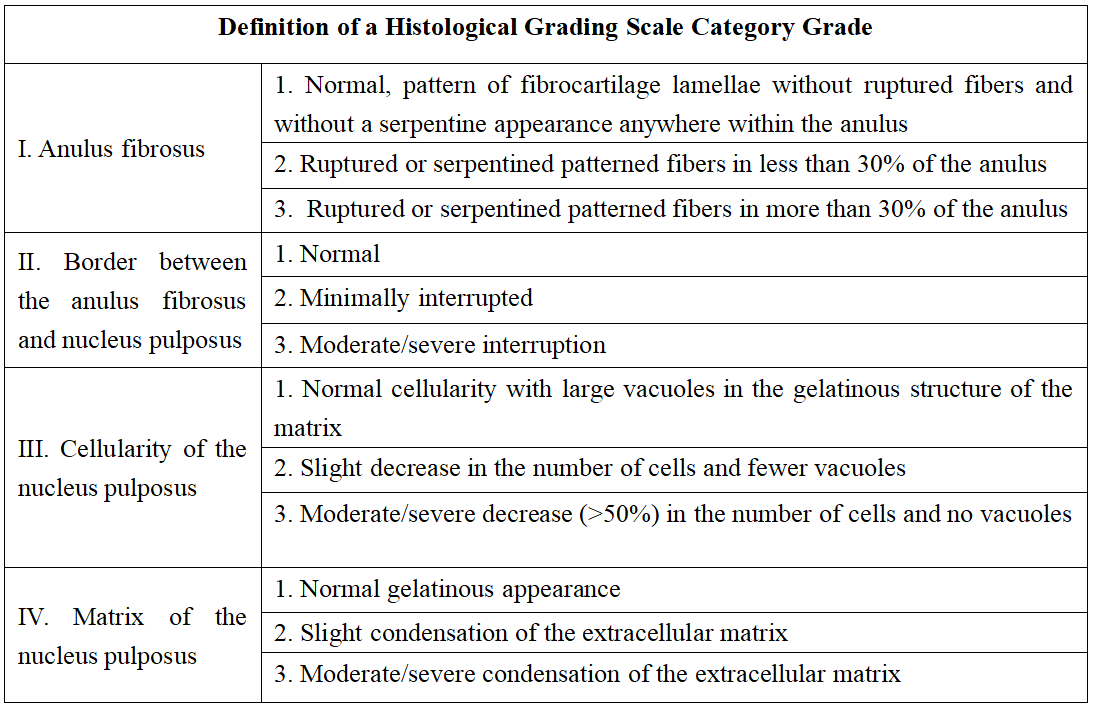


**Reference**

1. Van Der Spoel D, Lindahl E, Hess B, Groenhof G, Mark AE, Berendsen HJ. GROMACS: fast, flexible, and free. *J Comput Chem*. **2005** Dec;*26(16)*:1701-18.
2. Darden T, York D, Pedersen L. Particle Mesh Ewald: An *N*⋅log (N) Method for Ewald Sums in Large Systems. *J Chem Phys*. **1993**; *98(12)*:10089-10092.
3. DeLano, W L. Pymol: An Open-source Molecular Graphics Tool. *Pymol: An Open-source Molecular Graphics Tool*. **2002**; *40 (1)*: 82-92.
4. Trott O, Olson AJ. AutoDock Vina: Improving The Speed and Accuracy of Docking with A New Scoring Function, Efficient Optimization, and Multithreading. *J Comput Chem*. **2010**;*31(2)*: 455-61.
5. Peng Y, Chen X, Zhang Q, Liu S, Wu W, Li K, Lin H, Qing X, Xiao Y, Wang B, Quan D, Feng S, Rao Z, Bai Y, Shao Z. Enzymatically bioactive nucleus pulposus matrix hydrogel microspheres for exogenous stem cells therapy and endogenous repair strategy to achieve disc regeneration. *Adv Sci (Weinh)*. **2024** Mar;*11(10)*:e2304761.
6. Wang J, Huang Y, Luan T, Shi P, Guo L, Zhang Q, Shi G, Hao Z, Chen T, Zhang L, Li J. Hydrogel and Microgel Collaboration for Spatiotemporal Delivery of Biofactors to Awaken Nucleus Pulposus-Derived Stem Cells for Endogenous Repair of Disc. *Small*. **2024** Dec;*20(49)*:e2404732.
